# Supplementary material for: Genetically Predicted 25‐Hydroxyvitamin D Levels on Hypothyroidism: A Two‐Sample Mendelian Randomization
Source: Biomed Res Int. 2026 Jun 8;2026:8086558. doi: 10.1155/bmri/8086558 (PMC13244256; doi:10.1155/bmri/8086558)
Supplement: Supplementary file 1 — Supporting Information Additional supporting information can be found online in the Supporting Information section. 1 25‐Hydroxyvitamin D level_GCST90000616_buildGRCh37.tsv. This file contains the GWAS summary statistics for serum 25‐hydroxyvitamin D levels, which were used as the exposure dataset for the selection of genetic instrumental variables in the Mendelian randomization analyses. Supporting Information 2. Hypothyroidism_GCST90204167_buildGRCh37.tsv. This file contains the GWAS summary statistics for hypothyroidism, which were used as the primary outcome dataset to evaluate the causal effect of genetically predicted 25‐hydroxyvitamin D levels on hypothyroidism risk. Supporting Information 3. Hashimoto′s Disease_GCST90018855_buildGRCh37.tsv. This file contains the GWAS summary statistics for Hashimoto′s thyroiditis, which were used as an autoimmune thyroid disease outcome in the Mendelian randomization analyses. Supporting Information 4. TSH.xlsx. This file contains genetic association data for thyroid‐stimulating hormone (TSH), which were used to assess the association between genetically predicted 25‐hydroxyvitamin D levels and circulating TSH levels. Supporting Information 5. free T4.xlsx. This file contains genetic association data for free thyroxine (free T4), which were used to assess the association between genetically predicted 25‐hydroxyvitamin D levels and circulating free T4 levels. Supporting Information 6. Supplementary figures.docx. This file contains the supplementary graphical outputs from the Mendelian randomization analyses, including variant‐specific forest plots, leave‐one‐out analyses, funnel plots, comparisons of causal estimates across Mendelian randomization methods, and additional sensitivity analyses for hypothyroidism, Hashimoto′s thyroiditis, free T4, and TSH. [file BMRI-2026-8086558-s001.zip › supplemantry figures.docx]

Supplementary materials

Title: **Genetically predicted 25-Hydroxyvitamin D levels on Hypothyroidism: evidence from Mendelian randomization studies**

Running title: **Relationship between Vitamin D and Hypothyroidism**

**Authors:**

- 1. Mahdi Akbarzadeh; Ph.D., Cellular and Molecular Endocrine Research Center, Research Institute for Endocrine Sciences, Shahid Beheshti University of Medical Sciences, Tehran, Iran. Email: [akbarzadeh.ms@gmail.com](mailto:akbarzadeh.ms@gmail.com)
  2. Sahand Tehrani Fateh; MD, School of Medicine, Tehran University of Medical Sciences, Tehran, Iran. Email: [Sahh.tf@gmail.com](mailto:Sahh.tf@gmail.com)
  3. Aysan Moeinafshar, MD, School of Medicine, Tehran University of Medical Sciences, Tehran, Iran. Email: [aysanmoeinafshar@gmail.com](mailto:aysanmoeinafshar@gmail.com)
  4. Danial Habibi; Ph.D., Department of Epidemiology and Biostatistics, School of Public Health, Babol University of Medical Sciences, Babol, Iran. Email: [dhabibi67@gmail.com](mailto:dhabibi67@gmail.com)
  5. Amir Hossein Ghanooni; MD, Department of Endocrinology, School of Medicine, Iran University of Medical Sciences, Tehran, Iran. Email: [ircms@yahoo.com](mailto:ircms@yahoo.com)
  6. Amir Hesam Saeidian; MD, Department of Surgery, Rasool-E Akram Hospital, School of Medicine, Iran University of Medical Sciences, Tehran, Iran. Email:  [amirhesam_saeidian@yahoo.com](mailto:amirhesam_saeidian@yahoo.com)
  7. Parisa Riahi; MSc, Cellular and Molecular Endocrine Research Center, Research Institute for Endocrine Sciences, Shahid Beheshti University of Medical Sciences, Tehran, Iran. Email: [parisaariyahii@gmail.com](mailto:parisaariyahii@gmail.com)
  8. Maryam Zarkesh: Cellular and Molecular Endocrine Research Center, Research Institute for Endocrine Sciences, Shahid Beheshti University of Medical Sciences, Tehran, Iran. Email: [zarkesh@endocrine.ac.ir](mailto:zarkesh@endocrine.ac.ir)
  9. Hossein Lanjanian; Cellular and Molecular Endocrine Research Center, Research Institute for Endocrine Sciences, Shahid Beheshti University of Medical Sciences, Tehran, Iran. Email: [hossein.lanjanian@gmail.com](mailto:hossein.lanjanian@gmail.com)
  10. Mina Jahangiri; Ph.D., Department of Biostatistics, Faculty of Medical Sciences, Tarbiat Modares University, Tehran, Iran. Email: [minajahangiri984@gmail.com](mailto:minajahangiri984@gmail.com)
  11. Maryam Moazzam-Jazi; Ph.D, Cellular and Molecular Endocrine Research Center, Research Institute for Endocrine Sciences, Shahid Beheshti University of Medical Sciences, Tehran, Iran. Email: [setareh227@gmail.com](mailto:setareh227@gmail.com)
  12. Farshad Teymoori, Nutrition and Endocrine Research Center, Research Institute for Endocrine Sciences, Shahid Beheshti, Tehran, Iran. Email: [teymoori.f68@gmail.com](mailto:teymoori.f68@gmail.com)
  13. Fereidoun Azizi; MD, Endocrine Research Center, Research Institute for Endocrine Sciences, Shahid Beheshti University of Medical Sciences, Tehran, Iran. Email: [azizi@endocrine.ac.ir](mailto:azizi@endocrine.ac.ir)
  14. Mehdi Hedayati; Ph.D., Cellular and Molecular Endocrine Research Center, Research Institute for Endocrine Sciences, Shahid Beheshti University of Medical Sciences, Tehran, Iran. Email: [hedayati47@gmail.com](mailto:hedayati47@gmail.com)
  15. Maryam Sadat Daneshpour; Ph.D., Cellular and Molecular Endocrine Research Center, Research Institute for Endocrine Sciences, Shahid Beheshti University of Medical Sciences, Tehran, Iran. Email: [daneshpour@sbmu.ac.ir](mailto:daneshpour@sbmu.ac.ir)

**Corresponding author:**

Maryam Sadat Daneshpour (Ph.D.), Associate Professor.

Cellular and Molecular Endocrine Research Center, Research Institute for Endocrine Sciences, Shahid Beheshti University of Medical Sciences. Email: [daneshpour@sbmu.ac.ir](mailto:daneshpour@sbmu.ac.ir)

Tehran, Iran; PO Box: 19395-4763; Tel: +98(21)22432500; Fax: +98(21)22416264

**Table of content:**

**Figure S1**: Forest plot of variant specific inverse variance estimates for causal association between 25-Hydroxyvitamin D levels Hypothyroidism.

**Figure S2**: Leave-one-out plot to assess if a single variant is driving the association between 25-Hydroxyvitamin D levels Hypothyroidism.

**Figure S3**. Funnel plot of causal association between 25-Hydroxyvitamin D levels Hypothyroidism.

**Figure S4**: Comparison of the causal estimates from the various Mendelian randomization methods.

**Figure S5**: Sensitivity analyses.

**Figure S1**: Forest plot of variant specific inverse variance estimates for causal association between 25-Hydroxyvitamin D levels Hypothyroidism

A) Hypothyroidism


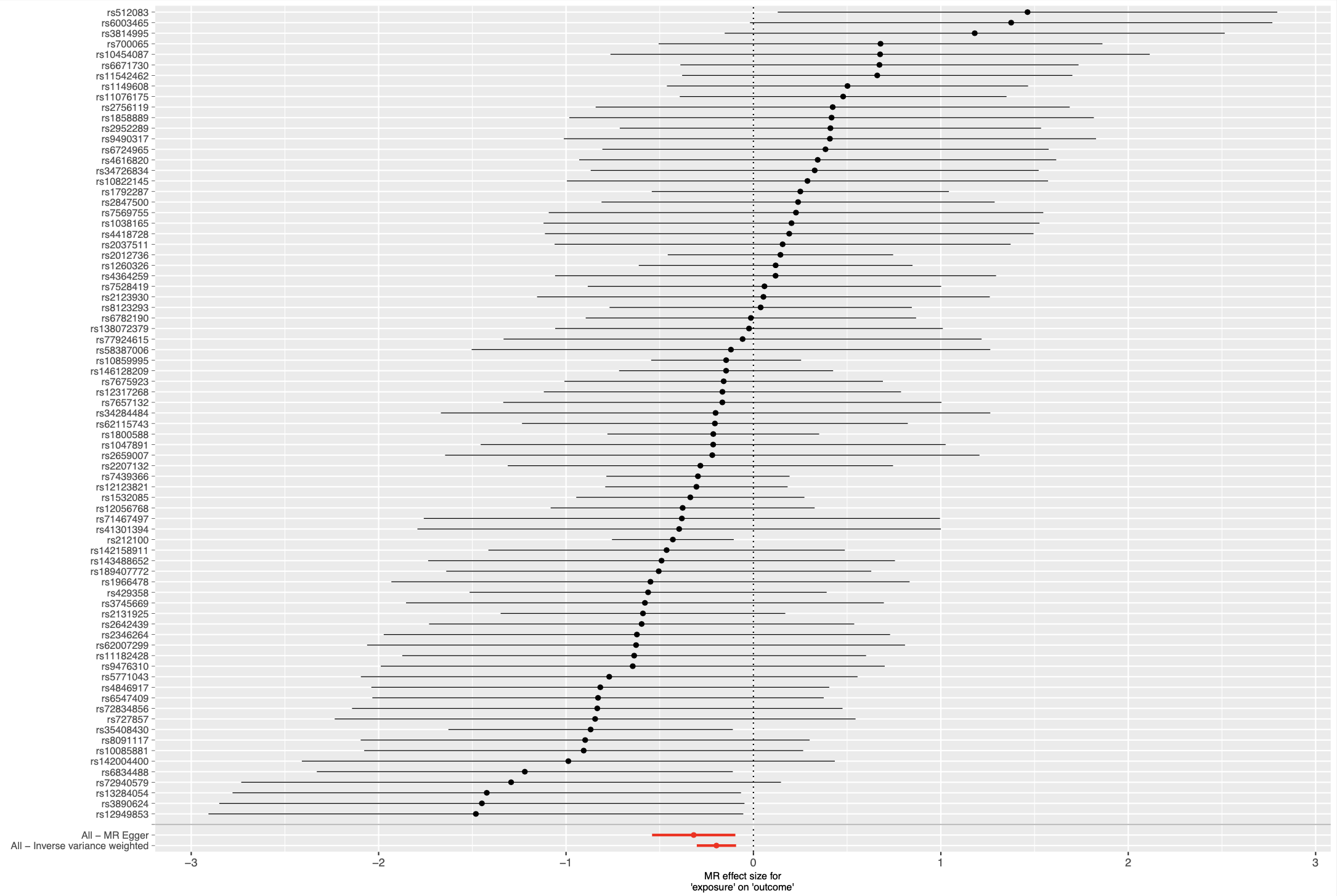


B) Hashimoto’s thyroiditis


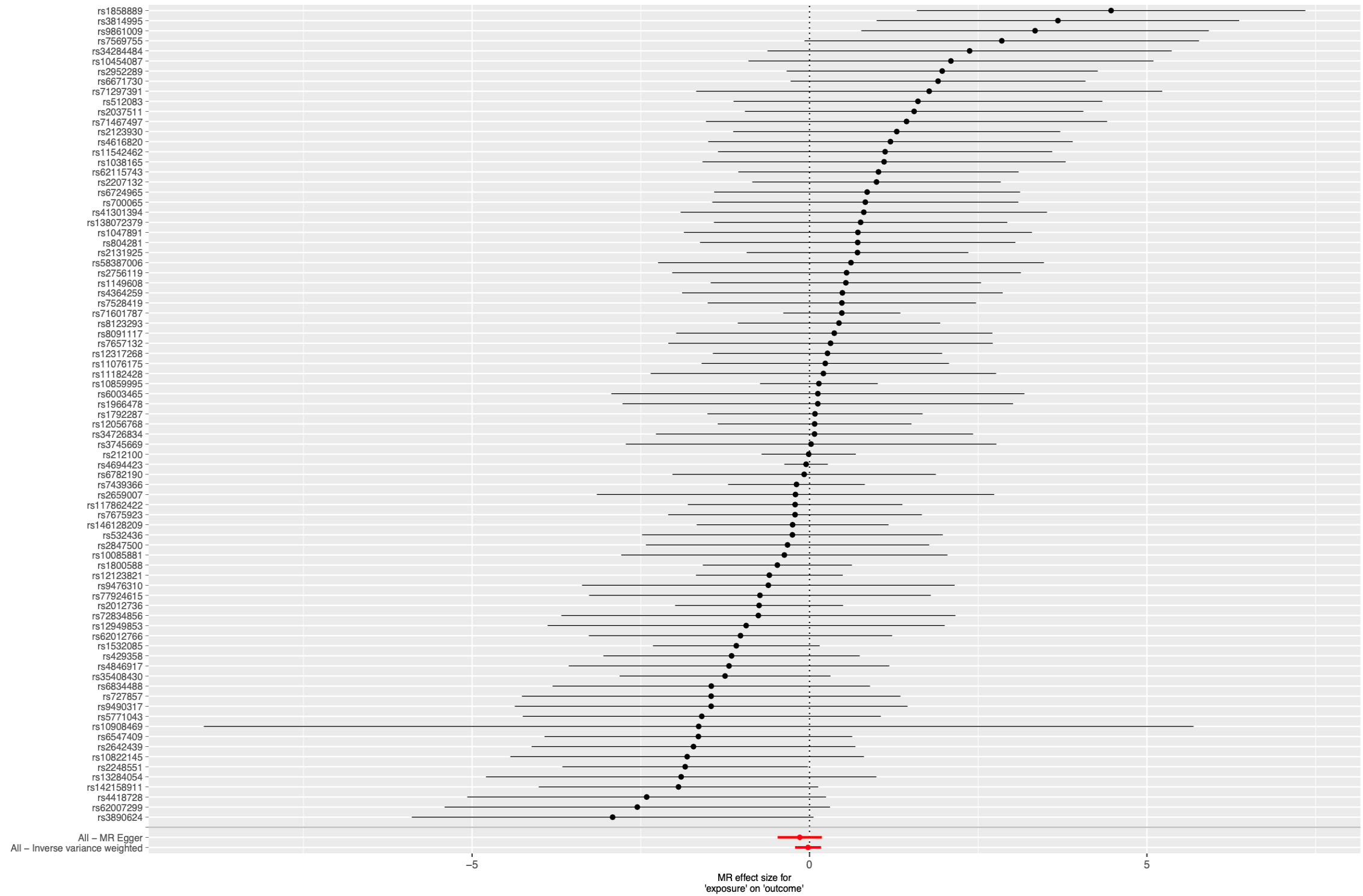


C) Free T4


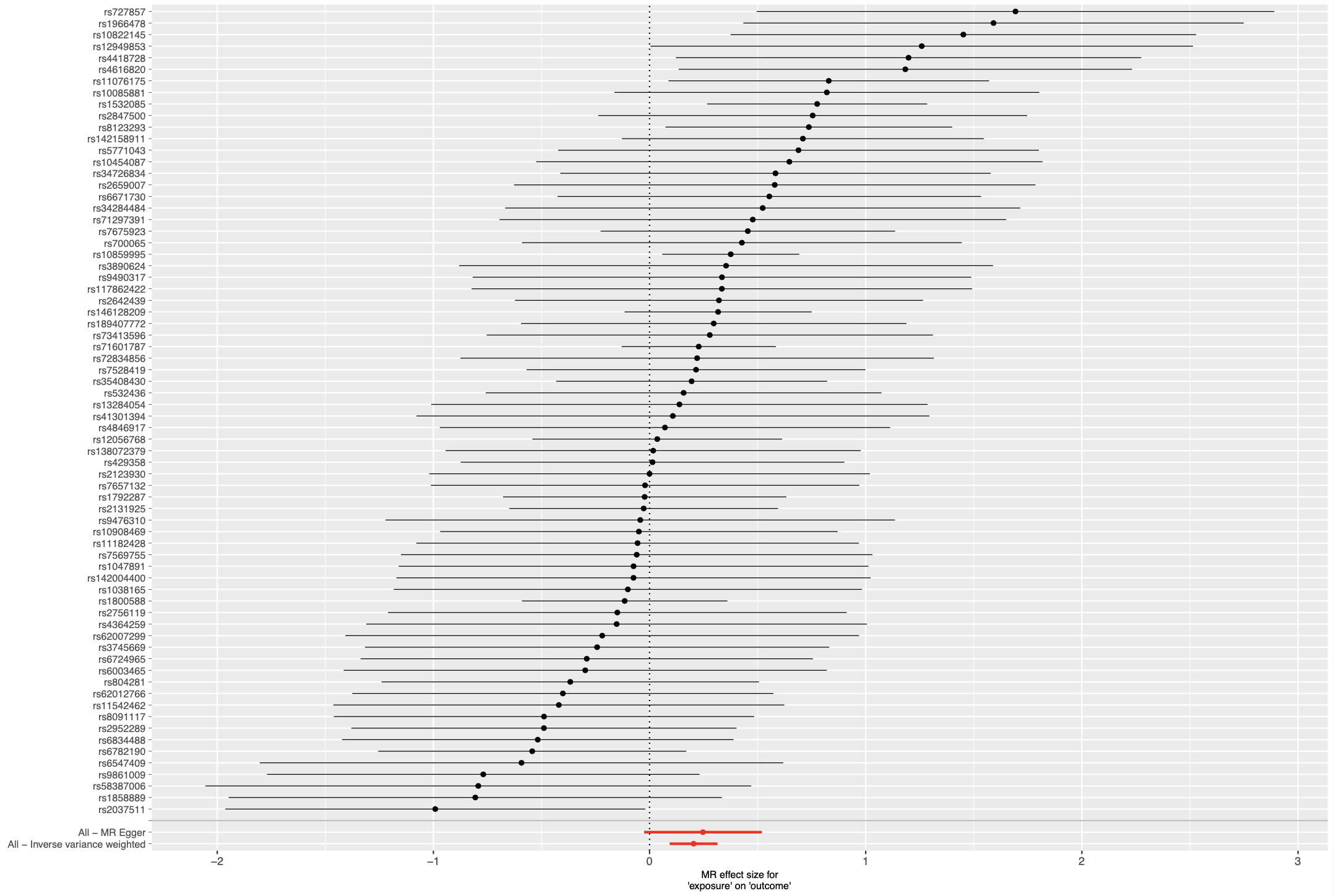


D) TSH


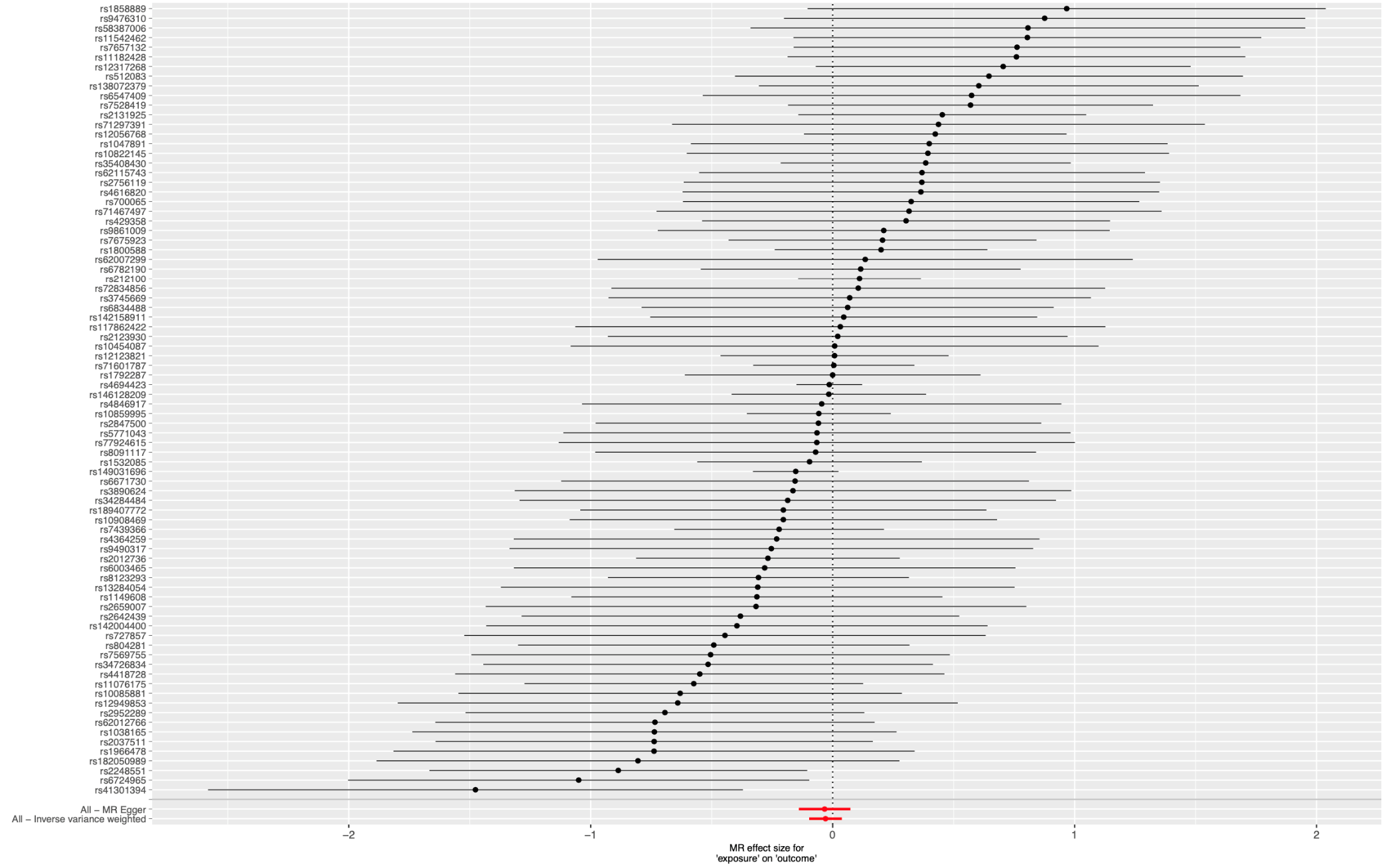


**Figure S2:** Leave-one-out plot to assess if a single variant is driving the association between 25-Hydroxyvitamin D levels Hypothyroidism

A) Hypothyroidism loo


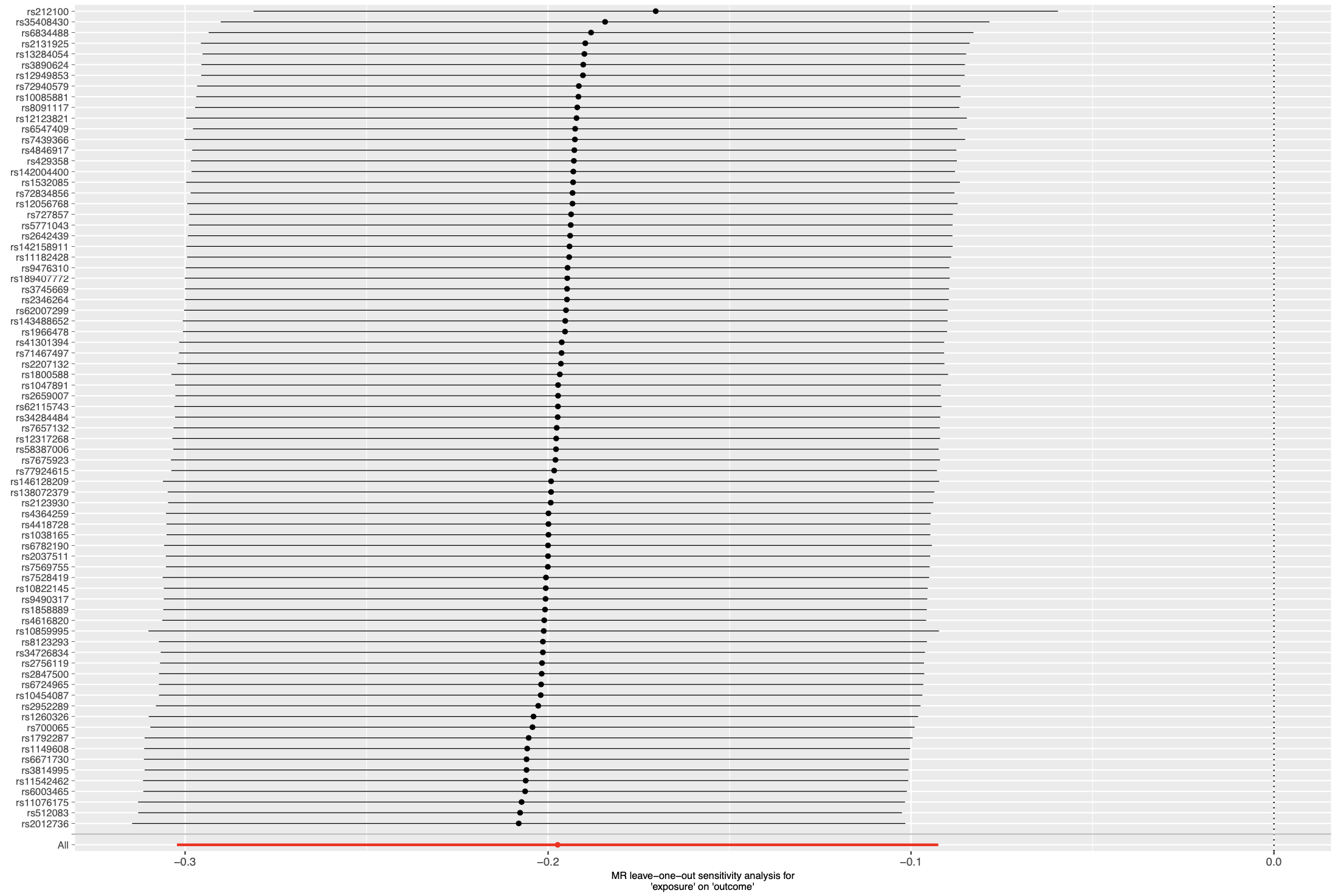


B) Hashimoto’s thyroiditis


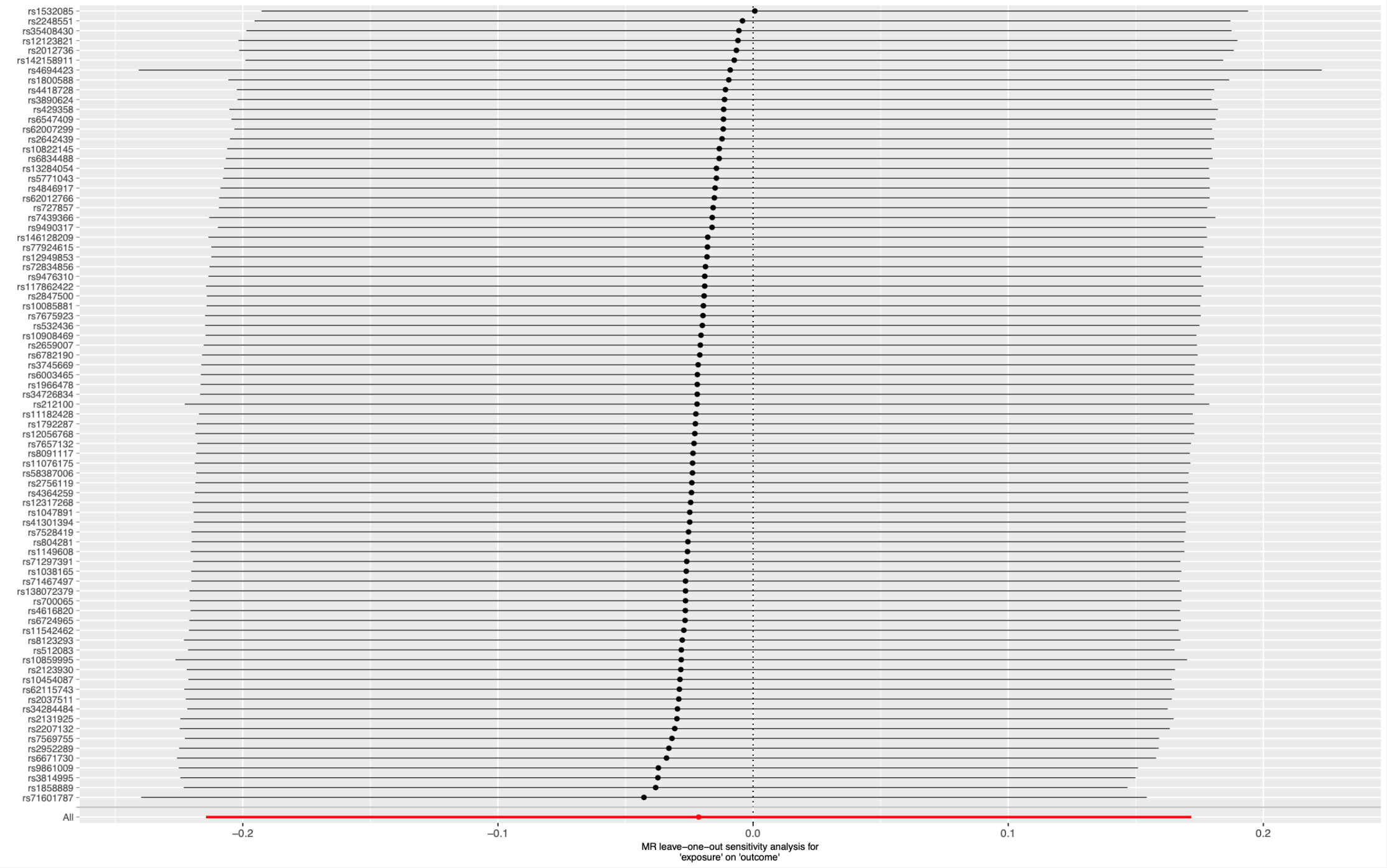


C) Free T4


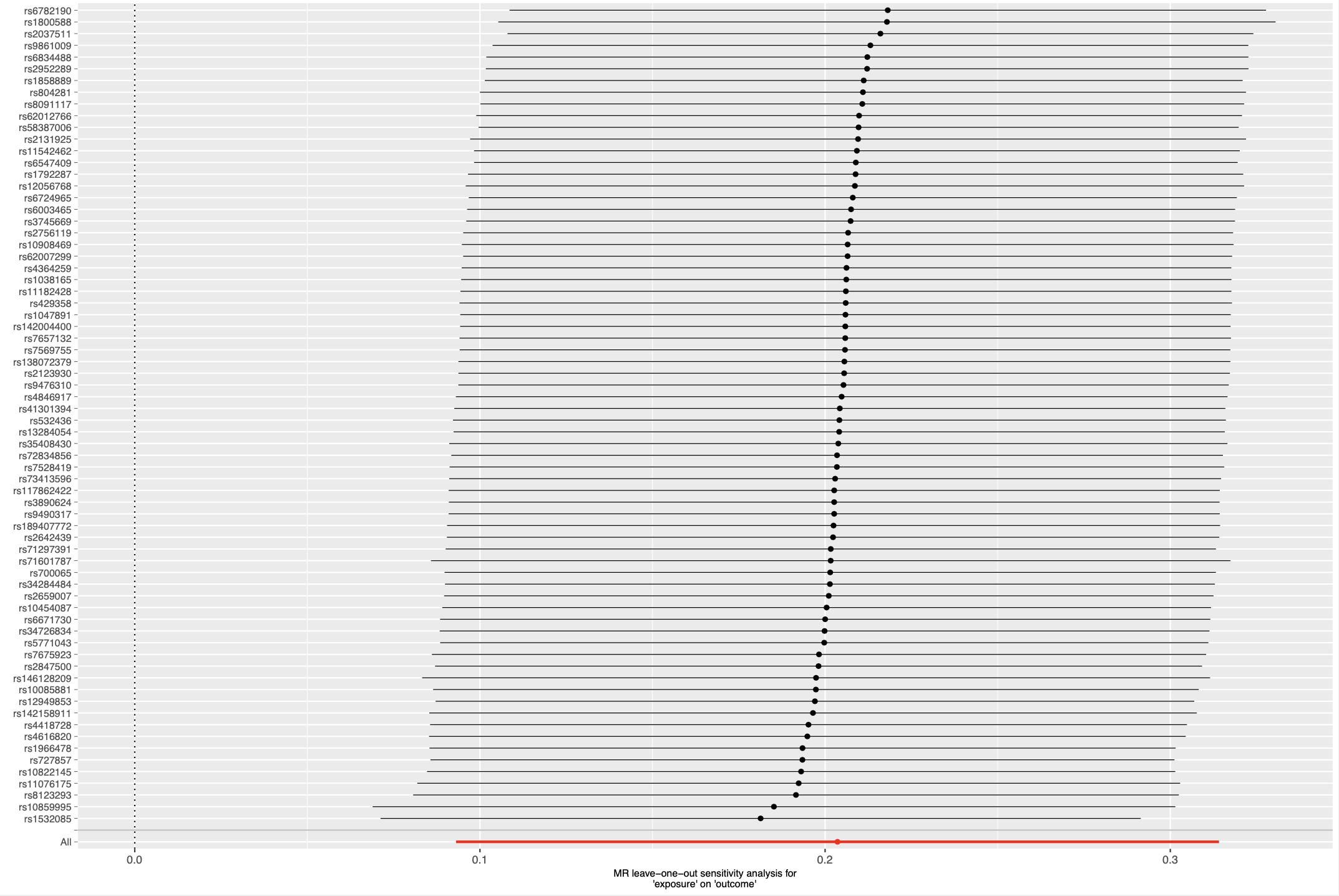


D) TSH


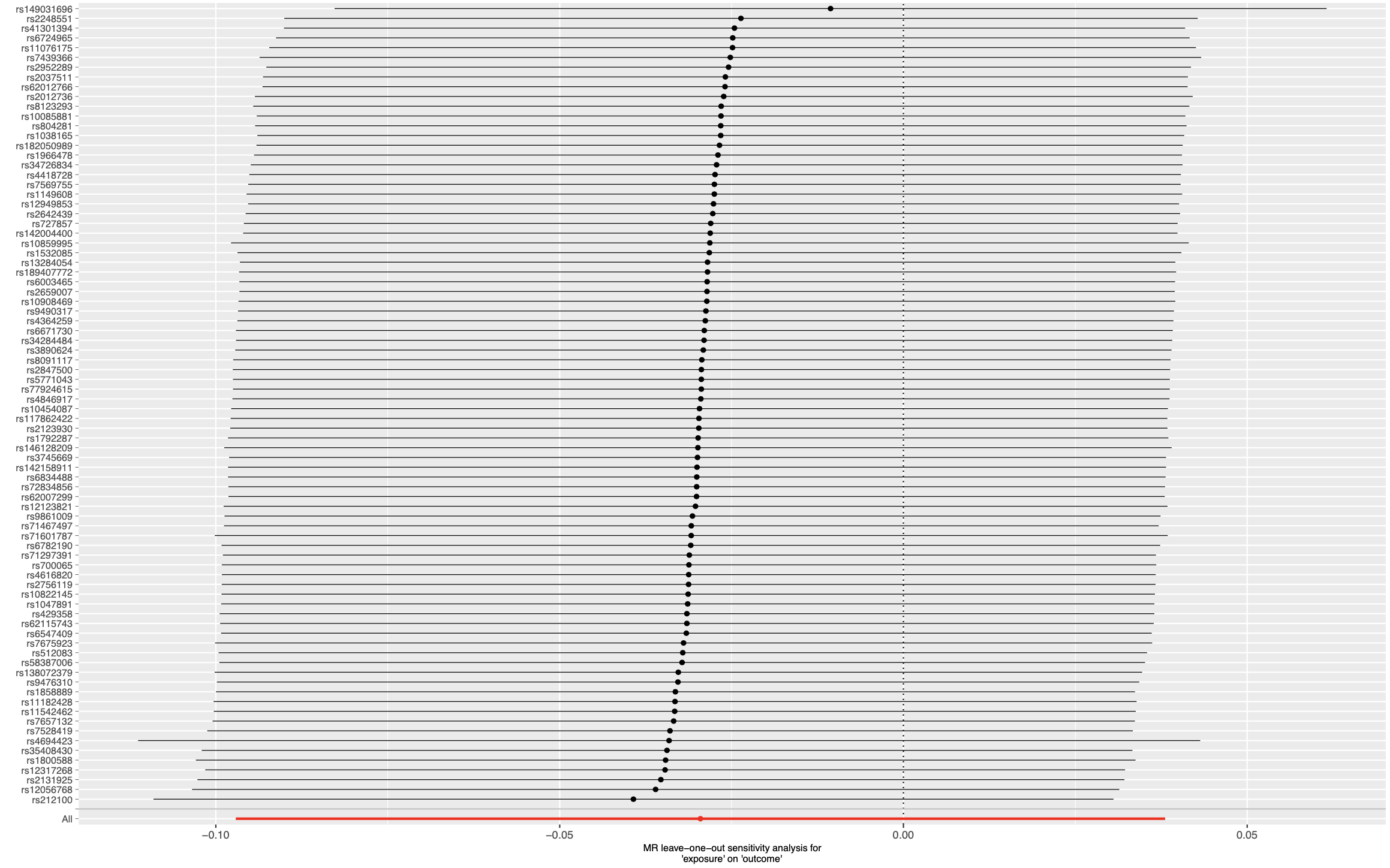


**Figure S3.** Funnel plot of causal association between 25-Hydroxyvitamin D levels Hypothyroidism

A) Hypothyroidism


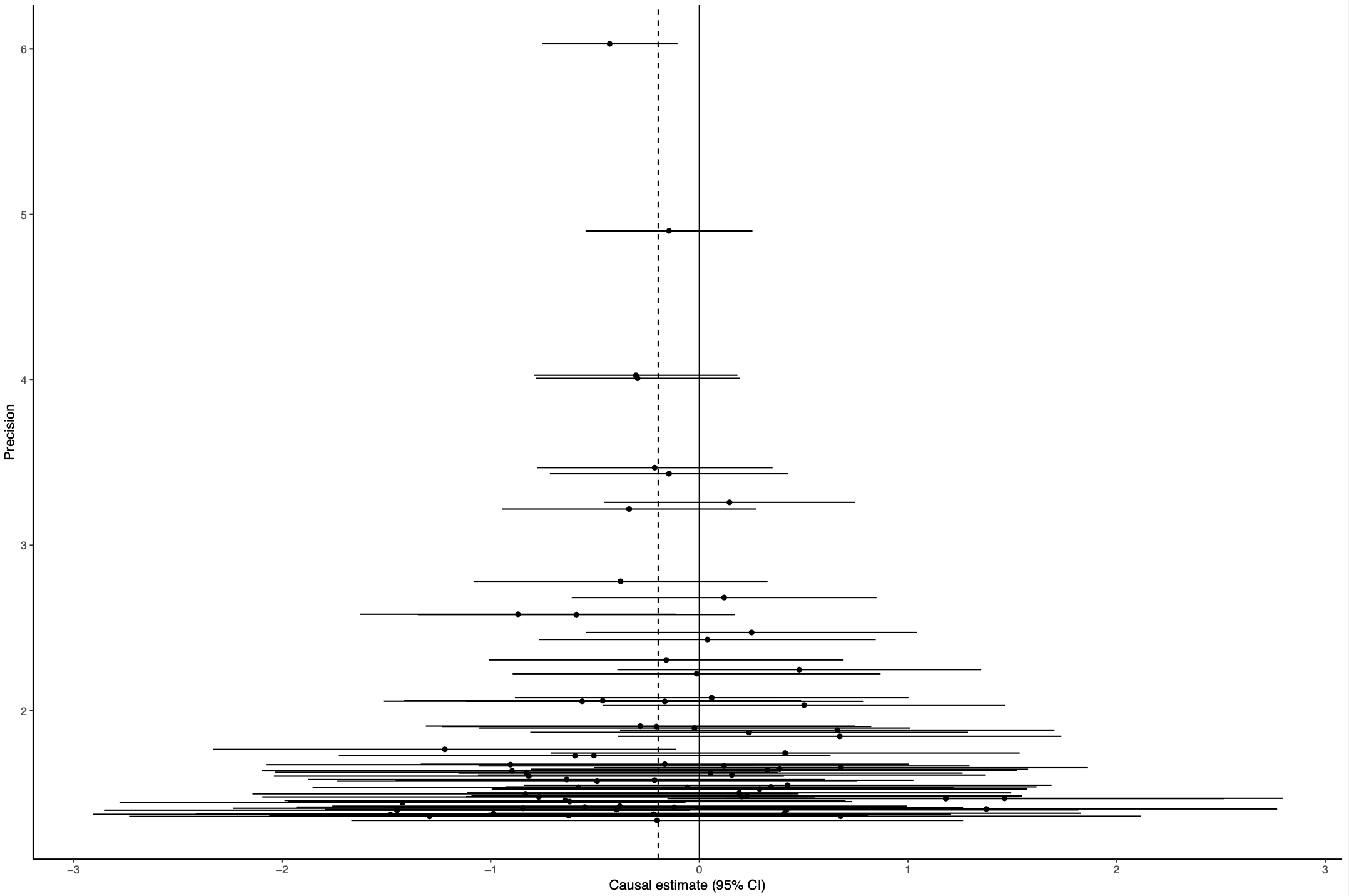


B) Hashimoto’s thyroiditis


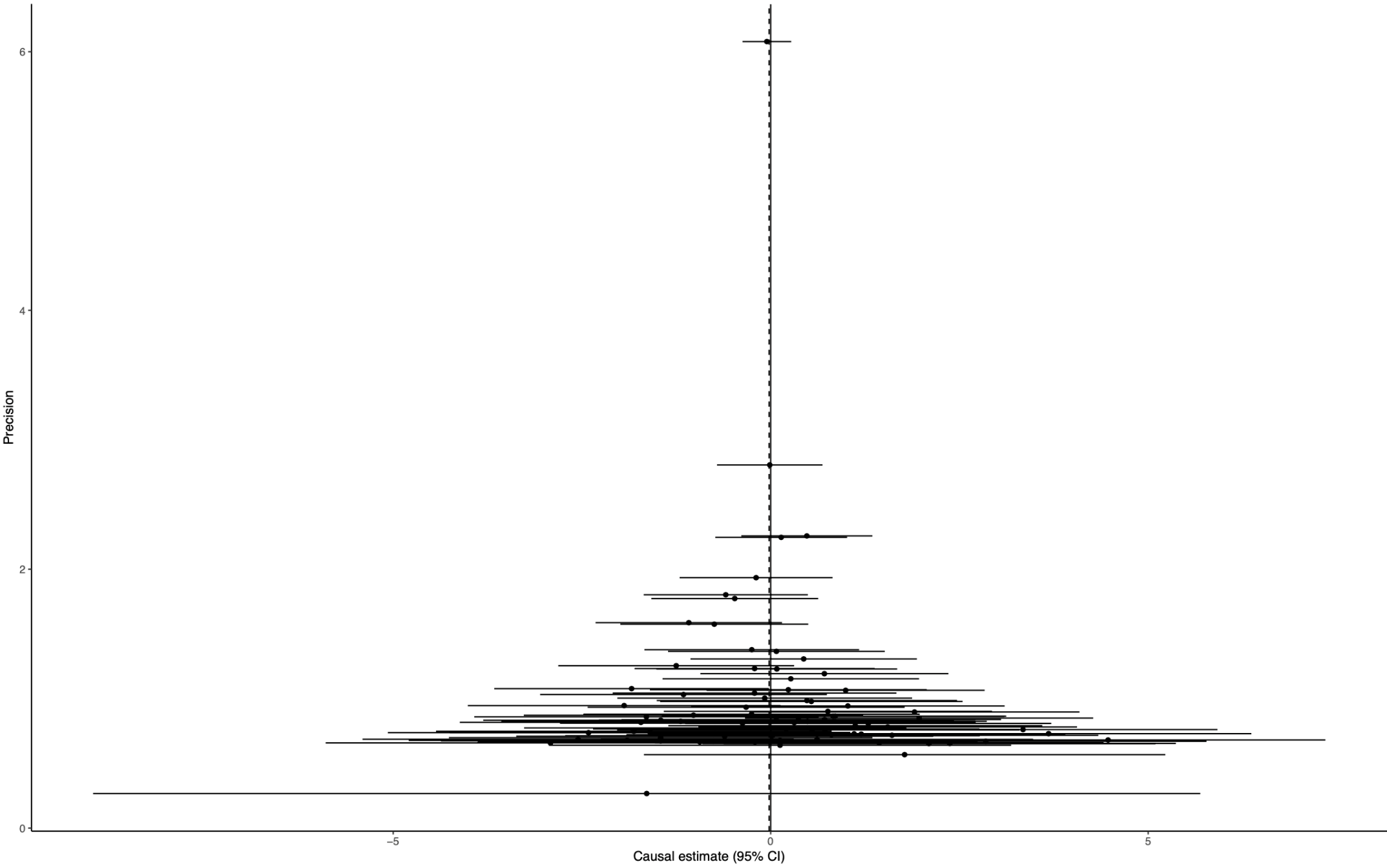


C) Free T4


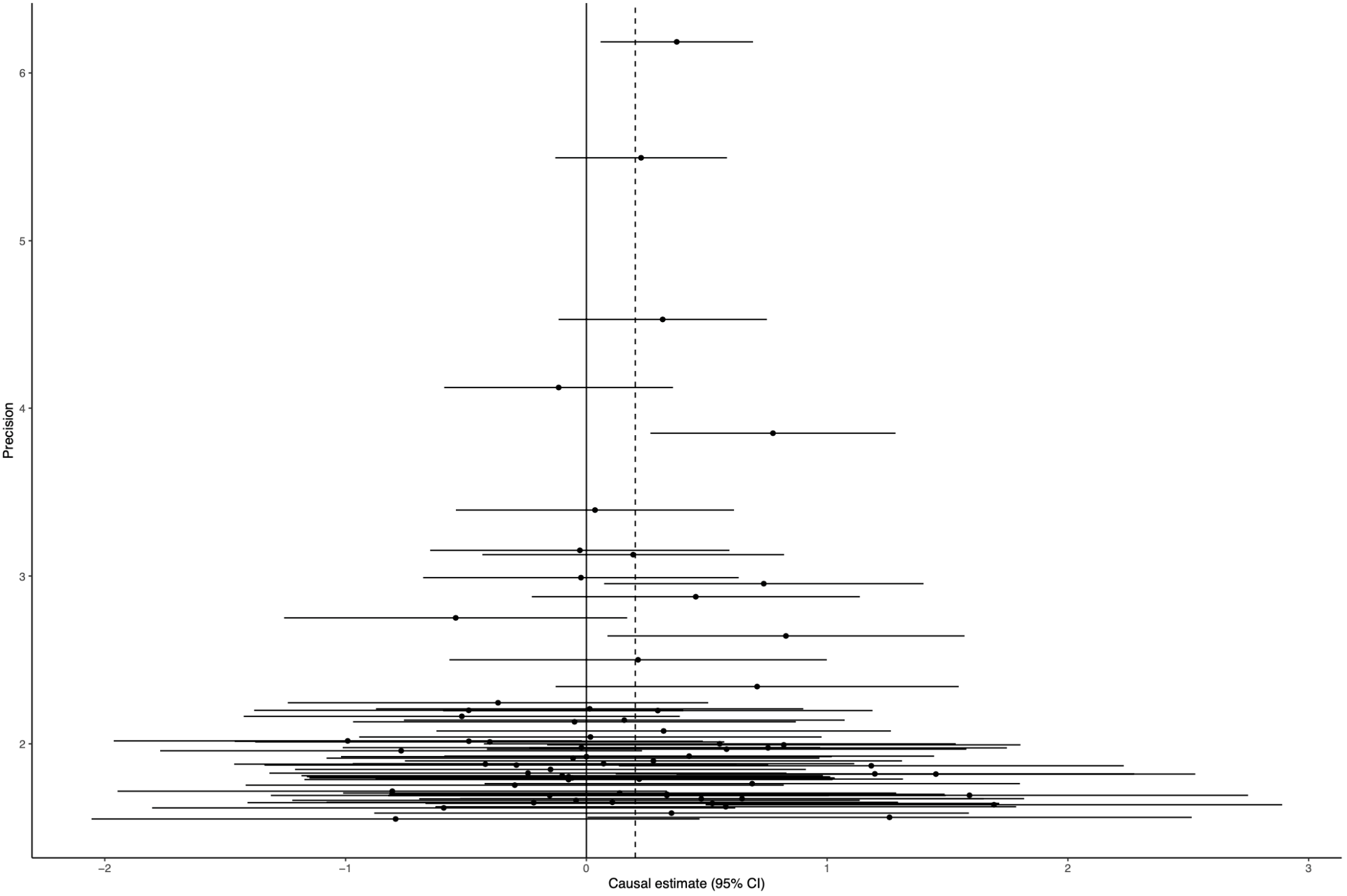


D) TSH


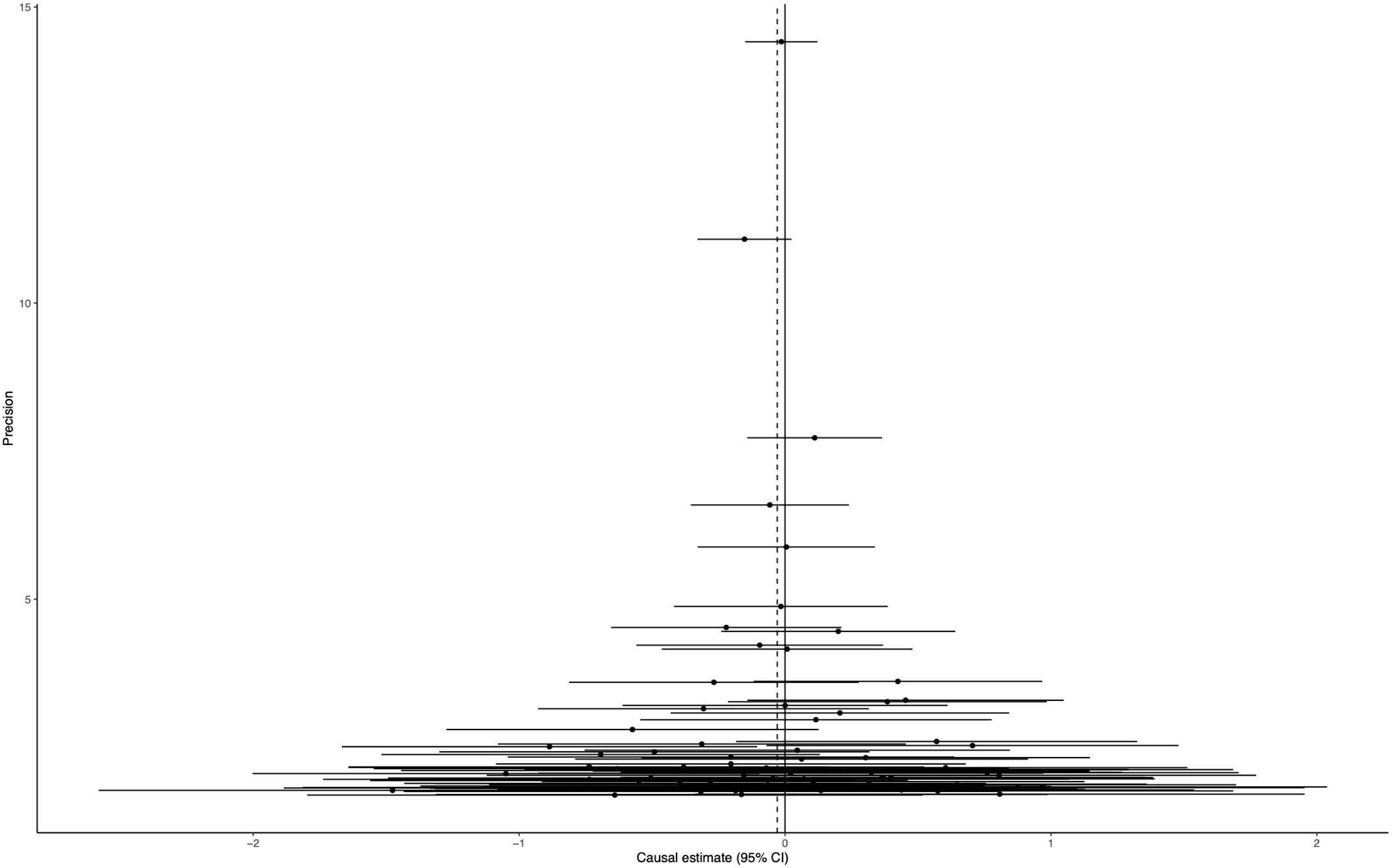


**Figure S4**: Comparison of the causal estimates from the various Mendelian randomization methods.


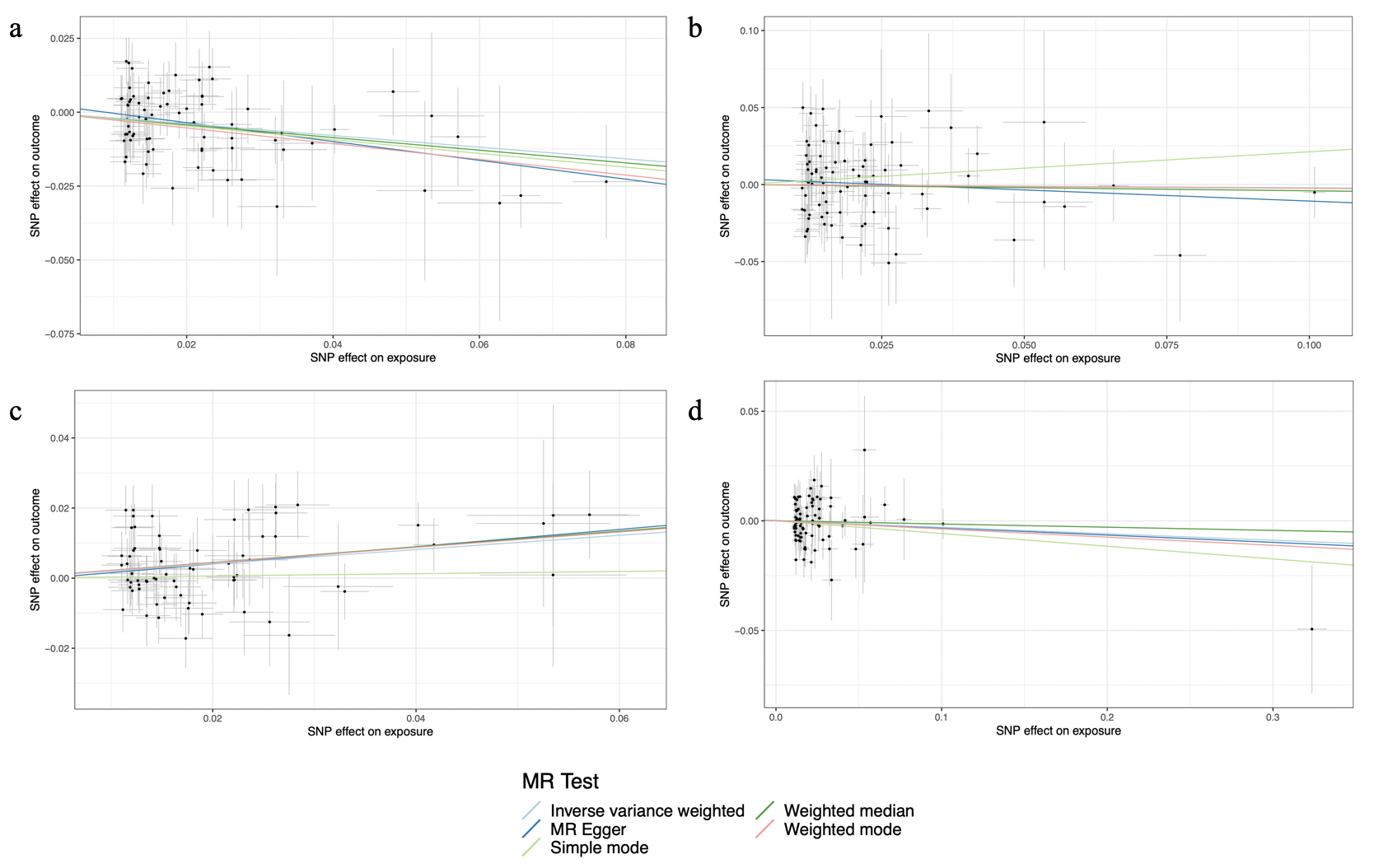


A: Hypothyroidism, B: Hashimoto's thyroiditis, C: Free T4 level, D: TSH level.

**Figure S5**: Sensitivity analyses.


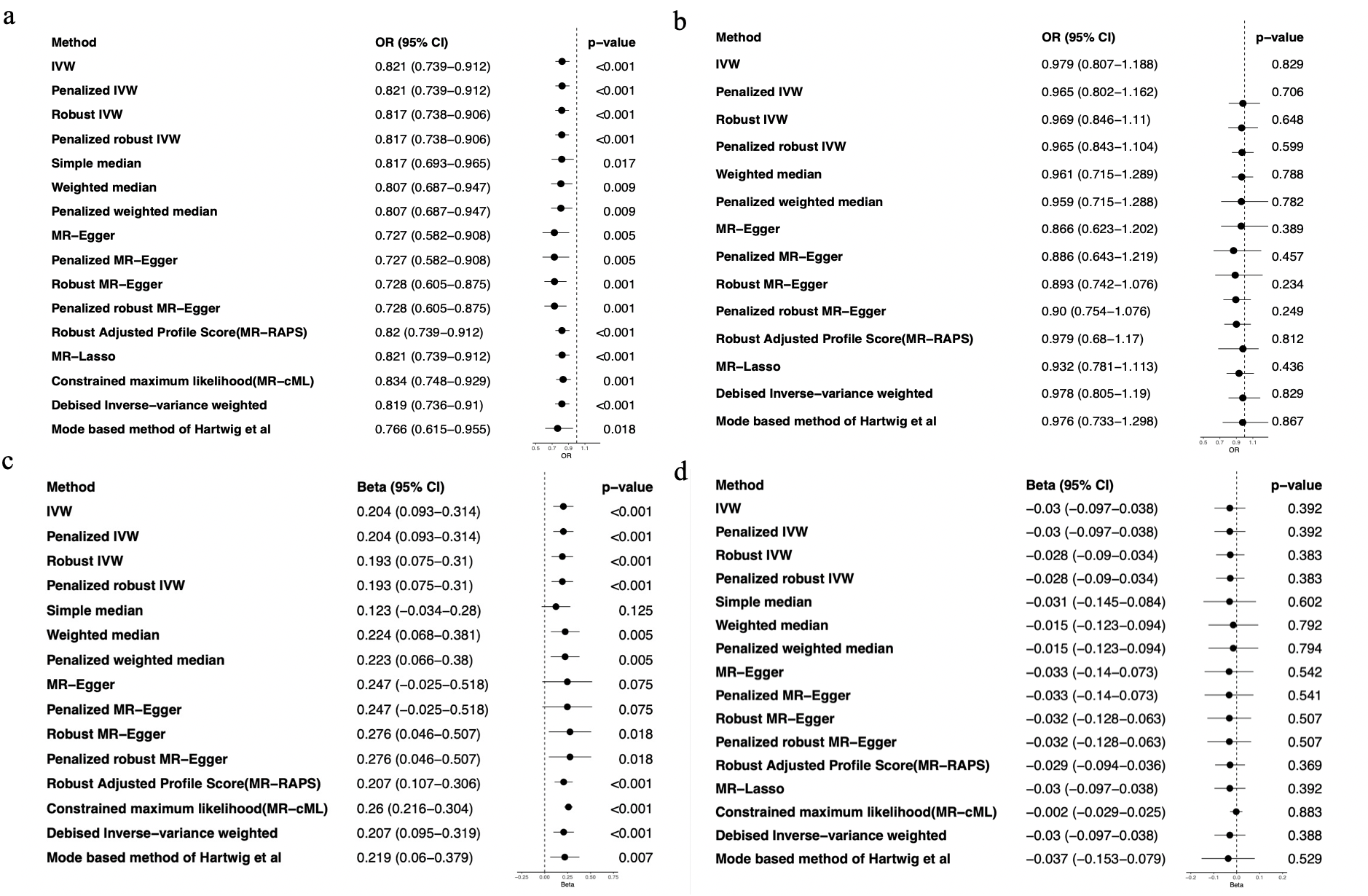


A: Hypothyroidism, B: Hashimoto's thyroiditis, C: Free T4 level, D: TSH level.
