# Supplementary material for: Genetically Predicted 25‐Hydroxyvitamin D Levels on Hypothyroidism: A Two‐Sample Mendelian Randomization
Source: Biomed Res Int. 2026 Jun 8;2026:8086558. doi: 10.1155/bmri/8086558 (PMC13244256; doi:10.1155/bmri/8086558)
Supplement: Supplementary file 1 — Supporting Information Additional supporting information can be found online in the Supporting Information section. 1 25‐Hydroxyvitamin D level_GCST90000616_buildGRCh37.tsv. This file contains the GWAS summary statistics for serum 25‐hydroxyvitamin D levels, which were used as the exposure dataset for the selection of genetic instrumental variables in the Mendelian randomization analyses. Supporting Information 2. Hypothyroidism_GCST90204167_buildGRCh37.tsv. This file contains the GWAS summary statistics for hypothyroidism, which were used as the primary outcome dataset to evaluate the causal effect of genetically predicted 25‐hydroxyvitamin D levels on hypothyroidism risk. Supporting Information 3. Hashimoto′s Disease_GCST90018855_buildGRCh37.tsv. This file contains the GWAS summary statistics for Hashimoto′s thyroiditis, which were used as an autoimmune thyroid disease outcome in the Mendelian randomization analyses. Supporting Information 4. TSH.xlsx. This file contains genetic association data for thyroid‐stimulating hormone (TSH), which were used to assess the association between genetically predicted 25‐hydroxyvitamin D levels and circulating TSH levels. Supporting Information 5. free T4.xlsx. This file contains genetic association data for free thyroxine (free T4), which were used to assess the association between genetically predicted 25‐hydroxyvitamin D levels and circulating free T4 levels. Supporting Information 6. Supplementary figures.docx. This file contains the supplementary graphical outputs from the Mendelian randomization analyses, including variant‐specific forest plots, leave‐one‐out analyses, funnel plots, comparisons of causal estimates across Mendelian randomization methods, and additional sensitivity analyses for hypothyroidism, Hashimoto′s thyroiditis, free T4, and TSH. [file BMRI-2026-8086558-s001.zip › VitD_Hypo_MR.html]

Mendelian Randomization Report [25-Hydroxyvitamin D level and Hypothyroidism]


# Mendelian Randomization Report [25-Hydroxyvitamin D level and Hypothyroidism]

#### Mahdi Akbarzadeh - Email: akbarzadeh.ms@gmail.com - mob: (+98)9126490780

#### Sahand Tehrani Fateh

#### 2023-08-29

# Genetically predicted 25-Hydroxyvitamin D levels on hypothyroidism: A two-sample Mendelian Randomization

1. Mahdi Akbarzadeh; Ph.D., Cellular and Molecular Endocrine Research
   Center, Research Institute for Endocrine Sciences, Shahid Beheshti
   University of Medical Sciences, Tehran, Iran. Email: akbarzadeh.ms@gmail.com
2. Sahand Tehrani Fateh; MD, School of Medicine, Tehran University of
   Medical Sciences, Tehran, Iran. Email: Sahh.tf@gmail.com
3. Aysan Moeinafshar, MD, School of Medicine, Tehran University of
   Medical Sciences, Tehran, Iran. Email: aysanmoeinafshar@gmail.com
4. Danial Habibi; Ph.D., Cellular and Molecular Endocrine Research
   Center, Research Institute for Endocrine Sciences, Shahid Beheshti
   University of Medical Sciences, Tehran, Iran. Email: dhabibi67@gmail.com
5. Amir Hossein Ghanooni; MD, Department of Endocrinology, School of
   Medicine, Iran University of Medical Sciences, Tehran, Iran. Email: ircms@yahoo.com
6. Hesam Saeidian; MD, Department of Surgery, Rasool-E Akram Hospital,
   School of Medicine, Iran University of Medical Sciences, Tehran, Iran.
   Email: amirhesam\_saeidian@yahoo.com
7. Parisa Riahi; MSc, Cellular and Molecular Endocrine Research Center,
   Research Institute for Endocrine Sciences, Shahid Beheshti University of
   Medical Sciences, Tehran, Iran. Email: parisaariyahii@gmail.com
8. Maryam Zarkesh: Cellular and Molecular Endocrine Research Center,
   Research Institute for Endocrine Sciences, Shahid Beheshti University of
   Medical Sciences, Tehran, Iran. Email: zarkesh@endocrine.ac.ir
9. Hossein Lanjanian; Cellular and Molecular Endocrine Research Center,
   Research Institute for Endocrine Sciences, Shahid Beheshti University of
   Medical Sciences, Tehran, Iran. Email: hossein.lanjanian@gmail.com
10. Mina Jahangiri; Ph.D., Department of Biostatistics, Faculty of
    Medical Sciences, Tarbiat, Modares University, Tehran, Iran. Email: minajahangiri984@gmail.com
11. Farshad Teymoori, Nutrition and Endocrine Research Center, Research
    Institute for Endocrine Sciences, Shahid Beheshti, Tehran, Iran. Email:
    teymoori.f68@gmail.com
12. Fereidoun Azizi; MD, Endocrine research center, Research Institute
    for Endocrine Sciences, Shahid Beheshti University of Medical Sciences,
    Tehran, Iran. Email: azizi@endocrine.ac.ir
13. Mehdi Hedayati; Ph.D., Cellular and Molecular Endocrine Research
    Center, Research Institute for Endocrine Sciences, Shahid Beheshti
    University of Medical Sciences, Tehran, Iran. Email: hedayati47@gmail.com
14. Maryam Sadat Daneshpour; Ph.D., Cellular and Molecular Endocrine
    Research Center, Research Institute for Endocrine Sciences, Shahid
    Beheshti University of Medical Sciences, Tehran, Iran. Email: daneshpour@sbmu.ac.ir

- **Abbreviations:**
  - **VitD :** 25-Hydroxyvitamin D level
  - **Hypo :** Hypothyroidism
  - **HT :** Hashimoto’s thyroiditis
  - **fT4 :** Free T4 level
  - **TSH :** TSH level

## [VitD and Hypo]

### Introduction

- **Title:** Investigating the causality between
  **25-Hydroxyvitamin D level on Hypothyroidism**

  - Exposure: 25-Hydroxyvitamin D level, Data downloaded from GWAS catalog
    Database | Reference paper: Joana A. Revez
    Paper 2020 | GWAS ID: GCST90000616
  - **Sample size**: 417,580
  - Outcome: Hypothyroidism, Data downloaded from GWAS catalog
    Database | Reference paper: Samuel Mathieu 2022
    | GWAS ID: GCST90204167
  - **Sample size**:494,577 , **Number of
    cases**:51,194 , **Number of controls**:
    443,383
  - Ancestry: **European**

### Data Preparation

1- **Number of total SNPs in exposure:** 7,250,104
SNPs

2- **Number of SNPs exposure with p-value <** \(5 \times 10^-8\): 16,012 SNPs

3- **Number of SNPs exposure after clumping** : 115
SNPs

4- **Number of total SNPs in outcome**: 10,836,150
SNPs

5- **Number of common variants between exposure and
outcome**: 109 SNPs

6- **Number of SNPs after harmonization (action=3)**  =
90 SNPs

7- **Number of SNPs after removing HLA region with exploring in
HLA Genes,
Nomenclature** = 89 SNP (rs28407950 was removed)

8- **Number of SNPs after removing those that have MAF <
0.01** = 89 SNPs

10- Checking pleiotropy by PhenoScanner:

How many SNPs have been eliminated after checking the PhenoScanner
website: 0 SNPs

### Checking weakness of the instruments

```
##    Min. 1st Qu.  Median    Mean 3rd Qu.    Max. 
##   29.78   36.13   42.63  117.54   70.06 2567.54
```

How many SNPs have been eliminated with checking the weakness: 0
SNP

### RUN an initial MR:

Initial MR analysis


| id.exposure | id.outcome | outcome | exposure | method | nsnp | b | se | pval |
| --- | --- | --- | --- | --- | --- | --- | --- | --- |
| oUw2e5 | avirfC | outcome | exposure | MR Egger | 89 | -0.1464337 | 0.0916311 | 0.1136513 |
| oUw2e5 | avirfC | outcome | exposure | Weighted median | 89 | -0.1237461 | 0.0655389 | 0.0590083 |
| oUw2e5 | avirfC | outcome | exposure | Inverse variance weighted | 89 | -0.1307693 | 0.0577112 | 0.0234559 |
| oUw2e5 | avirfC | outcome | exposure | Simple mode | 89 | -0.1865331 | 0.1412272 | 0.1899920 |
| oUw2e5 | avirfC | outcome | exposure | Weighted mode | 89 | -0.1418811 | 0.0600942 | 0.0204351 |

Heterogeneity testing


| id.exposure | id.outcome | outcome | exposure | method | Q | Q\_df | Q\_pval |
| --- | --- | --- | --- | --- | --- | --- | --- |
| oUw2e5 | avirfC | outcome | exposure | MR Egger | 180.7067 | 87 | 0 |
| oUw2e5 | avirfC | outcome | exposure | Inverse variance weighted | 180.8081 | 88 | 0 |

pleiotropy testing


| id.exposure | id.outcome | outcome | exposure | egger\_intercept | se | pval |
| --- | --- | --- | --- | --- | --- | --- |
| oUw2e5 | avirfC | outcome | exposure | 0.0005249 | 0.0023765 | 0.8257019 |

### Testing Outlier with PRESSO test

```
## [1] "Two SNPs (rs73413596 and rs9861009) were detected by MRPRESSO and excluded for further analyses"
```

MR analysis after excluding SNPs detected by MRPRESSO


| id.exposure | id.outcome | outcome | exposure | method | nsnp | b | se | pval |
| --- | --- | --- | --- | --- | --- | --- | --- | --- |
| oUw2e5 | avirfC | outcome | exposure | MR Egger | 87 | -0.1334385 | 0.0800126 | 0.0990519 |
| oUw2e5 | avirfC | outcome | exposure | Weighted median | 87 | -0.1247436 | 0.0657183 | 0.0576754 |
| oUw2e5 | avirfC | outcome | exposure | Inverse variance weighted | 87 | -0.1267930 | 0.0505315 | 0.0121008 |
| oUw2e5 | avirfC | outcome | exposure | Simple mode | 87 | -0.1848303 | 0.1389260 | 0.1868961 |
| oUw2e5 | avirfC | outcome | exposure | Weighted mode | 87 | -0.1337747 | 0.0610113 | 0.0310351 |

Heterogeneity testing


| id.exposure | id.outcome | outcome | exposure | method | Q | Q\_df | Q\_pval |
| --- | --- | --- | --- | --- | --- | --- | --- |
| oUw2e5 | avirfC | outcome | exposure | MR Egger | 134.4047 | 85 | 0.0005139 |
| oUw2e5 | avirfC | outcome | exposure | Inverse variance weighted | 134.4230 | 86 | 0.0006569 |

pleiotropy testing


| id.exposure | id.outcome | outcome | exposure | egger\_intercept | se | pval |
| --- | --- | --- | --- | --- | --- | --- |
| oUw2e5 | avirfC | outcome | exposure | 0.0002243 | 0.0020857 | 0.9146166 |

### Studentized residuals:

### Radial test

```
## 
## Radial IVW
## 
##                    Estimate  Std.Error   t value    Pr(>|t|)
## Effect (Mod.2nd) -0.1267937 0.05053137 -2.509208 0.012100225
## Iterative        -0.1267937 0.05053137 -2.509208 0.012100225
## Exact (FE)       -0.1284882 0.04043783 -3.177427 0.001485882
## Exact (RE)       -0.1278726 0.05679221 -2.251586 0.026896844
## 
## 
## Residual standard error: 1.25 on 86 degrees of freedom
## 
## F-statistic: 6.3 on 1 and 86 DF, p-value: 0.014
## Q-Statistic for heterogeneity: 134.2939 on 86 DF , p-value: 0.0006739595
## 
##  No significant outliers 
## Number of iterations = 2
```

```
## [1] "No significant outliers"
```

### Cook’s distance

In statistics, Cook’s distance or Cook’s D is a commonly used
estimate of the influence of a data point when performing a
least-squares regression analysis.[1] In a practical ordinary least
squares analysis, Cook’s distance can be used in several ways:

1- To indicate influential data points that are particularly worth
checking for validity.

2- To indicate regions of the design space where it would be good to
be able to obtain more data points.

**It is named after the American statistician R. Dennis Cook,
who introduced the concept in 1977.**

Refernce

```
##        23        82 
## 7.9211985 0.3927332
```

### Run After deleting new outlier: Final Results:

MR analysis after deleting outliers


| id.exposure | id.outcome | outcome | exposure | method | nsnp | b | se | pval |
| --- | --- | --- | --- | --- | --- | --- | --- | --- |
| oUw2e5 | avirfC | outcome | exposure | MR Egger | 77 | -0.3186386 | 0.1133876 | 0.0063124 |
| oUw2e5 | avirfC | outcome | exposure | Weighted median | 77 | -0.2144203 | 0.0808106 | 0.0079693 |
| oUw2e5 | avirfC | outcome | exposure | Inverse variance weighted | 77 | -0.1973681 | 0.0535110 | 0.0002257 |
| oUw2e5 | avirfC | outcome | exposure | Simple mode | 77 | -0.2324364 | 0.1595762 | 0.1493499 |
| oUw2e5 | avirfC | outcome | exposure | Weighted mode | 77 | -0.2661283 | 0.1057838 | 0.0139871 |

Heterogeneity testing


| id.exposure | id.outcome | outcome | exposure | method | Q | Q\_df | Q\_pval |
| --- | --- | --- | --- | --- | --- | --- | --- |
| oUw2e5 | avirfC | outcome | exposure | MR Egger | 73.88599 | 75 | 0.5147087 |
| oUw2e5 | avirfC | outcome | exposure | Inverse variance weighted | 75.35763 | 76 | 0.4992441 |

pleiotropy testing


| id.exposure | id.outcome | outcome | exposure | egger\_intercept | se | pval |
| --- | --- | --- | --- | --- | --- | --- |
| oUw2e5 | avirfC | outcome | exposure | 0.002823 | 0.0023271 | 0.2288948 |

### Sensitivity analyses with MendelianRandomization Package

```
## 
## Inverse-variance weighted method
## (variants uncorrelated, random-effect model)
## 
## Number of Variants : 77 
## 
## ------------------------------------------------------------------
##  Method Estimate Std Error  95% CI        p-value
##     IVW   -0.197     0.054 -0.302, -0.092   0.000
## ------------------------------------------------------------------
## Residual standard error =  0.996 
## Residual standard error is set to 1 in calculation of confidence interval when its estimate is less than 1.
## Heterogeneity test statistic (Cochran's Q) = 75.3576 on 76 degrees of freedom, (p-value = 0.4992). I^2 = 0.0%.
```

```
##                     Method Estimate Std Error 95% CI         P-value
##              Simple median   -0.202     0.084  -0.367 -0.036   0.017
##            Weighted median   -0.215     0.082  -0.375 -0.054   0.009
##  Penalized weighted median   -0.215     0.082  -0.375 -0.054   0.009
##                                                                     
##                        IVW   -0.197     0.054  -0.302 -0.092   0.000
##              Penalized IVW   -0.197     0.054  -0.302 -0.092   0.000
##                 Robust IVW   -0.202     0.052  -0.304 -0.099   0.000
##       Penalized robust IVW   -0.202     0.052  -0.304 -0.099   0.000
##                                                                     
##                   MR-Egger   -0.319     0.113  -0.541 -0.096   0.005
##                (intercept)    0.003     0.002  -0.002  0.007   0.225
##         Penalized MR-Egger   -0.319     0.113  -0.541 -0.096   0.005
##                (intercept)    0.003     0.002  -0.002  0.007   0.225
##            Robust MR-Egger   -0.317     0.094  -0.502 -0.133   0.001
##                (intercept)    0.003     0.002  -0.002  0.007   0.230
##  Penalized robust MR-Egger   -0.317     0.094  -0.502 -0.133   0.001
##                (intercept)    0.003     0.002  -0.002  0.007   0.230
```

| id.exposure | id.outcome | exposure | outcome | snp\_r2.exposure | snp\_r2.outcome | correct\_causal\_direction | steiger\_pval |
| --- | --- | --- | --- | --- | --- | --- | --- |
| oUw2e5 | avirfC | exposure | outcome | 0.0138834 | 0.0001799 | TRUE | 0 |

```
## $r2_exp
## [1] 0
## 
## $r2_out
## [1] 0.25
## 
## $r2_exp_adj
## [1] 0
## 
## $r2_out_adj
## [1] 0.25
## 
## $correct_causal_direction
## [1] FALSE
## 
## $steiger_test
## [1] 0
## 
## $correct_causal_direction_adj
## [1] FALSE
## 
## $steiger_test_adj
## [1] 0
## 
## $vz
## [1] NaN
## 
## $vz0
## [1] 0
## 
## $vz1
## [1] NaN
## 
## $sensitivity_ratio
## [1] NaN
## 
## $sensitivity_plot
```

### Working with MRraps

```
## $beta.hat
## [1] -0.1999762
## 
## $beta.se
## [1] 0.05431763
## 
## $beta.p.value
## [1] 0.0002317672
## 
## $naive.se
## [1] 0.05395171
## 
## $chi.sq.test
## [1] 75.17799
```

```
##   over.dispersion loss.function   beta.hat    beta.se
## 1           FALSE            l2 -0.1999762 0.05431763
## 2           FALSE         huber -0.2055908 0.05573633
## 3           FALSE         tukey -0.2049427 0.05573554
## 4            TRUE            l2 -0.1999772 0.05432156
## 5            TRUE         huber -0.2056785 0.05588018
## 6            TRUE         tukey -0.2050190 0.05627405
```

```
## 
## MR-Lasso method 
## 
## Number of variants : 77 
## Number of valid instruments : 77 
## Tuning parameter : 0.2793058 
## ------------------------------------------------------------------
##  Exposure Estimate Std Error  95% CI        p-value
##  exposure   -0.197     0.054 -0.302, -0.092   0.000
## ------------------------------------------------------------------
```

```
## 
## Constrained maximum likelihood method (MRcML) 
## Number of Variants:  77 
## Results for:  cML-MA-BIC 
## ------------------------------------------------------------------
##      Method Estimate    SE Pvalue          95% CI
##  cML-MA-BIC   -0.200 0.054  0.000 [-0.306,-0.095]
## ------------------------------------------------------------------
```

```
## 
## Debiased inverse-variance weighted method
## (Over.dispersion:TRUE)
## 
## Number of Variants : 77 
## ------------------------------------------------------------------
##  Method Estimate Std Error  95% CI        p-value Condition
##    dIVW   -0.200     0.054 -0.307, -0.094   0.000   652.186
## ------------------------------------------------------------------
```

```
## 
## Mode-based method of Hartwig et al
## (weighted, delta standard errors [not assuming NOME], bandwidth factor = 1)
## 
## Number of Variants : 77 
## ------------------------------------------------------------------
##  Method Estimate Std Error  95% CI        p-value
##     MBE   -0.266     0.112 -0.486, -0.046   0.018
## ------------------------------------------------------------------
```

## [VitD and HT]

### Introduction

- **Title:** Investigating the causality between
  **25-Hydroxyvitamin D level on Hashimoto’s Disease**

  - Exposure: 25-Hydroxyvitamin D level, Data downloaded from GWAS catalog
    Database | Reference paper: Joana A. Revez
    Paper 2020 | GWAS ID: GCST90000616
  - **Sample size**: 417,580
  - Outcome: Hashimoto’s Disease, Data downloaded from GWAS catalog
    Database | Reference paper: Saori Sakaue
    2021 | GWAS ID: GCST90018855
  - **Sample size**:395,640 , **Number of
    cases**:15,654 , **Number of controls**:
    379,986
  - Ancestry: **European**

### Data Preparation

1- **Number of total SNPs in exposure:** 7,250,104
SNPs

2- **Number of SNPs exposure with p-value <** \(5 \times 10^-8\): 16,012 SNPs

3- **Number of SNPs exposure after clumping** : 115
SNPs

4- **Number of total SNPs in outcome**: 25,797,652
SNPs

5- **Number of common variants between exposure and
outcome**: 106 SNPs

6- **Number of SNPs after harmonization (action=3)**  =
88 SNPs

7- **Number of SNPs after removing HLA region with exploring in
HLA Genes,
Nomenclature** = 87 SNP (rs28407950 was removed)

8- **Number of SNPs after removing those that have MAF <
0.01** = 87 SNPs

10- Checking pleiotropy by PhenoScanner:

How many SNPs have been eliminated after checking the PhenoScanner
website: 0 SNPs

### Checking weakness of the instruments

```
##    Min. 1st Qu.  Median    Mean 3rd Qu.    Max. 
##   29.78   35.78   43.13  118.55   67.82 2567.54
```

How many SNPs have been eliminated with checking the weakness: 0
SNP

### RUN an initial MR:

Initial MR analysis


| id.exposure | id.outcome | outcome | exposure | method | nsnp | b | se | pval |
| --- | --- | --- | --- | --- | --- | --- | --- | --- |
| oUw2e5 | jVh3lS | outcome | exposure | MR Egger | 87 | -0.1767241 | 0.1667552 | 0.2922463 |
| oUw2e5 | jVh3lS | outcome | exposure | Weighted median | 87 | -0.0450173 | 0.1496184 | 0.7635052 |
| oUw2e5 | jVh3lS | outcome | exposure | Inverse variance weighted | 87 | -0.0469765 | 0.1008700 | 0.6414211 |
| oUw2e5 | jVh3lS | outcome | exposure | Simple mode | 87 | 0.1921724 | 0.2910624 | 0.5108613 |
| oUw2e5 | jVh3lS | outcome | exposure | Weighted mode | 87 | -0.0462505 | 0.1292475 | 0.7213366 |

Heterogeneity testing


| id.exposure | id.outcome | outcome | exposure | method | Q | Q\_df | Q\_pval |
| --- | --- | --- | --- | --- | --- | --- | --- |
| oUw2e5 | jVh3lS | outcome | exposure | MR Egger | 112.7475 | 85 | 0.0237107 |
| oUw2e5 | jVh3lS | outcome | exposure | Inverse variance weighted | 114.0142 | 86 | 0.0233072 |

pleiotropy testing


| id.exposure | id.outcome | outcome | exposure | egger\_intercept | se | pval |
| --- | --- | --- | --- | --- | --- | --- |
| oUw2e5 | jVh3lS | outcome | exposure | 0.004073 | 0.0041678 | 0.3312166 |

### Testing Outlier with PRESSO test

```
## [1] "Two SNPs (rs73413596 and rs9861009) were detected by MRPRESSO and excluded for further analyses"
```

MR analysis after excluding SNPs detected by MRPRESSO


| id.exposure | id.outcome | outcome | exposure | method | nsnp | b | se | pval |
| --- | --- | --- | --- | --- | --- | --- | --- | --- |
| oUw2e5 | jVh3lS | outcome | exposure | MR Egger | 87 | -0.1767241 | 0.1667552 | 0.2922463 |
| oUw2e5 | jVh3lS | outcome | exposure | Weighted median | 87 | -0.0450173 | 0.1507754 | 0.7652666 |
| oUw2e5 | jVh3lS | outcome | exposure | Inverse variance weighted | 87 | -0.0469765 | 0.1008700 | 0.6414211 |
| oUw2e5 | jVh3lS | outcome | exposure | Simple mode | 87 | 0.1921724 | 0.2637881 | 0.4682802 |
| oUw2e5 | jVh3lS | outcome | exposure | Weighted mode | 87 | -0.0462505 | 0.1322257 | 0.7273557 |

Heterogeneity testing


| id.exposure | id.outcome | outcome | exposure | method | Q | Q\_df | Q\_pval |
| --- | --- | --- | --- | --- | --- | --- | --- |
| oUw2e5 | jVh3lS | outcome | exposure | MR Egger | 112.7475 | 85 | 0.0237107 |
| oUw2e5 | jVh3lS | outcome | exposure | Inverse variance weighted | 114.0142 | 86 | 0.0233072 |

pleiotropy testing


| id.exposure | id.outcome | outcome | exposure | egger\_intercept | se | pval |
| --- | --- | --- | --- | --- | --- | --- |
| oUw2e5 | jVh3lS | outcome | exposure | 0.004073 | 0.0041678 | 0.3312166 |

### Studentized residuals:

### Radial test

```
## 
## Radial IVW
## 
##                     Estimate  Std.Error    t value  Pr(>|t|)
## Effect (Mod.2nd) -0.04697674 0.10087009 -0.4657153 0.6414193
## Iterative        -0.04697674 0.10087009 -0.4657153 0.6414193
## Exact (FE)       -0.04752580 0.08760697 -0.5424887 0.5874819
## Exact (RE)       -0.04738330 0.07905517 -0.5993700 0.5505019
## 
## 
## Residual standard error: 1.151 on 86 degrees of freedom
## 
## F-statistic: 0.22 on 1 and 86 DF, p-value: 0.643
## Q-Statistic for heterogeneity: 114.0109 on 86 DF , p-value: 0.02331904
## 
##  No significant outliers 
## Number of iterations = 2
```

```
## [1] "No significant outliers"
```

### Cook’s distance

In statistics, Cook’s distance or Cook’s D is a commonly used
estimate of the influence of a data point when performing a
least-squares regression analysis.[1] In a practical ordinary least
squares analysis, Cook’s distance can be used in several ways:

1- To indicate influential data points that are particularly worth
checking for validity.

2- To indicate regions of the design space where it would be good to
be able to obtain more data points.

**It is named after the American statistician R. Dennis Cook,
who introduced the concept in 1977.**

Refernce

```
##        22        26        28        81 
## 2.2775207 0.1161799 0.3026865 0.3128092
```

### Run After deleting new outlier: Final Results:

MR analysis after deleting outliers


| id.exposure | id.outcome | outcome | exposure | method | nsnp | b | se | pval |
| --- | --- | --- | --- | --- | --- | --- | --- | --- |
| oUw2e5 | jVh3lS | outcome | exposure | MR Egger | 81 | -0.1444301 | 0.1675495 | 0.3912886 |
| oUw2e5 | jVh3lS | outcome | exposure | Weighted median | 81 | -0.0405204 | 0.1499723 | 0.7870171 |
| oUw2e5 | jVh3lS | outcome | exposure | Inverse variance weighted | 81 | -0.0213403 | 0.0985213 | 0.8285153 |
| oUw2e5 | jVh3lS | outcome | exposure | Simple mode | 81 | 0.2127241 | 0.2935103 | 0.4707148 |
| oUw2e5 | jVh3lS | outcome | exposure | Weighted mode | 81 | -0.0244143 | 0.1287613 | 0.8500957 |

Heterogeneity testing


| id.exposure | id.outcome | outcome | exposure | method | Q | Q\_df | Q\_pval |
| --- | --- | --- | --- | --- | --- | --- | --- |
| oUw2e5 | jVh3lS | outcome | exposure | MR Egger | 95.32294 | 79 | 0.1019003 |
| oUw2e5 | jVh3lS | outcome | exposure | Inverse variance weighted | 96.31948 | 80 | 0.1032066 |

pleiotropy testing


| id.exposure | id.outcome | outcome | exposure | egger\_intercept | se | pval |
| --- | --- | --- | --- | --- | --- | --- |
| oUw2e5 | jVh3lS | outcome | exposure | 0.0037247 | 0.0040985 | 0.3662284 |

### Sensitivity analyses with MendelianRandomization Package

```
## 
## Inverse-variance weighted method
## (variants uncorrelated, random-effect model)
## 
## Number of Variants : 81 
## 
## ------------------------------------------------------------------
##  Method Estimate Std Error  95% CI       p-value
##     IVW   -0.021     0.099 -0.214, 0.172   0.829
## ------------------------------------------------------------------
## Residual standard error =  1.097 
## Heterogeneity test statistic (Cochran's Q) = 96.3195 on 80 degrees of freedom, (p-value = 0.1032). I^2 = 16.9%.
```

```
##                     Method Estimate Std Error 95% CI        P-value
##              Simple median    0.081     0.155  -0.223 0.386   0.600
##            Weighted median   -0.040     0.150  -0.335 0.254   0.788
##  Penalized weighted median   -0.042     0.150  -0.336 0.253   0.782
##                                                                    
##                        IVW   -0.021     0.099  -0.214 0.172   0.829
##              Penalized IVW   -0.036     0.095  -0.221 0.150   0.706
##                 Robust IVW   -0.032     0.069  -0.167 0.104   0.648
##       Penalized robust IVW   -0.036     0.069  -0.171 0.099   0.599
##                                                                    
##                   MR-Egger   -0.144     0.168  -0.473 0.184   0.389
##                (intercept)    0.004     0.004  -0.004 0.012   0.363
##         Penalized MR-Egger   -0.121     0.163  -0.441 0.198   0.457
##                (intercept)    0.003     0.004  -0.005 0.011   0.499
##            Robust MR-Egger   -0.113     0.095  -0.298 0.073   0.234
##                (intercept)    0.003     0.004  -0.005 0.010   0.485
##  Penalized robust MR-Egger   -0.105     0.091  -0.283 0.073   0.249
##                (intercept)    0.002     0.004  -0.005 0.010   0.533
```

| id.exposure | id.outcome | exposure | outcome | snp\_r2.exposure | snp\_r2.outcome | correct\_causal\_direction | steiger\_pval |
| --- | --- | --- | --- | --- | --- | --- | --- |
| oUw2e5 | jVh3lS | exposure | outcome | 0.0171032 | 0.0002433 | TRUE | 0 |

```
## $r2_exp
## [1] 0
## 
## $r2_out
## [1] 0.25
## 
## $r2_exp_adj
## [1] 0
## 
## $r2_out_adj
## [1] 0.25
## 
## $correct_causal_direction
## [1] FALSE
## 
## $steiger_test
## [1] 0
## 
## $correct_causal_direction_adj
## [1] FALSE
## 
## $steiger_test_adj
## [1] 0
## 
## $vz
## [1] NaN
## 
## $vz0
## [1] 0
## 
## $vz1
## [1] NaN
## 
## $sensitivity_ratio
## [1] NaN
## 
## $sensitivity_plot
```

### Working with MRraps

```
## $beta.hat
## [1] -0.02158423
## 
## $beta.se
## [1] 0.09058503
## 
## $beta.p.value
## [1] 0.8116671
## 
## $naive.se
## [1] 0.0901482
## 
## $chi.sq.test
## [1] 96.31883
```

```
##   over.dispersion loss.function    beta.hat    beta.se
## 1           FALSE            l2 -0.02158423 0.09058503
## 2           FALSE         huber -0.03384769 0.09293915
## 3           FALSE         tukey -0.03399085 0.09293933
## 4            TRUE            l2 -0.02255761 0.09770638
## 5            TRUE         huber -0.03664930 0.10136241
## 6            TRUE         tukey -0.03232759 0.10111534
```

```
## 
## MR-Lasso method 
## 
## Number of variants : 81 
## Number of valid instruments : 78 
## Tuning parameter : 0.2830687 
## ------------------------------------------------------------------
##  Exposure Estimate Std Error  95% CI       p-value
##  exposure   -0.070     0.090 -0.247, 0.107   0.436
## ------------------------------------------------------------------
```

```
## 
## Constrained maximum likelihood method (MRcML) 
## Number of Variants:  81 
## Results for:  cML-MA-BIC 
## ------------------------------------------------------------------
##      Method Estimate    SE Pvalue         95% CI
##  cML-MA-BIC   -0.024 0.091  0.788 [-0.202,0.153]
## ------------------------------------------------------------------
```

```
## 
## Debiased inverse-variance weighted method
## (Over.dispersion:TRUE)
## 
## Number of Variants : 81 
## ------------------------------------------------------------------
##  Method Estimate Std Error  95% CI       p-value Condition
##    dIVW   -0.022     0.100 -0.217, 0.174   0.829   968.842
## ------------------------------------------------------------------
```

```
## 
## Mode-based method of Hartwig et al
## (weighted, delta standard errors [not assuming NOME], bandwidth factor = 1)
## 
## Number of Variants : 81 
## ------------------------------------------------------------------
##  Method Estimate Std Error  95% CI       p-value
##     MBE   -0.024     0.146 -0.310, 0.261   0.867
## ------------------------------------------------------------------
```

## [VitD and fT4]

### Introduction

- **Title:** Investigating the causality between
  **25-Hydroxyvitamin D level on fT4**

  - Exposure: 25-Hydroxyvitamin D level, Data downloaded from GWAS catalog
    Database | Reference paper: Joana A. Revez
    Paper 2020 | GWAS ID: GCST90000616
  - **Sample size**: 417,580
  - Outcome: fT4, Data downloaded from Thyroid
    Omics Consortium | Reference paper: Alexander
    Teumer 2018 | GWAS ID: phs000930
  - **Sample size**:49,269
  - Ancestry: **European**

### Data Preparation

1- **Number of total SNPs in exposure:** 7,250,104
SNPs

2- **Number of SNPs exposure with p-value <** \(5 \times 10^-8\): 16,012 SNPs

3- **Number of SNPs exposure after clumping** : 115
SNPs

4- **Number of total SNPs in outcome**: 7,745,739
SNPs

5- **Number of common variants between exposure and
outcome**: 101 SNPs

6- **Number of SNPs after harmonization (action=3)**  =
84 SNPs

7- **Number of SNPs after removing HLA region with exploring in
HLA Genes,
Nomenclature** = 84 SNP (rs28407950 was removed)

8- **Number of SNPs after removing those that have MAF <
0.01** = 84 SNPs

10- Checking pleiotropy by PhenoScanner:

How many SNPs have been eliminated after checking the PhenoScanner
website: 0 SNPs

### Checking weakness of the instruments

```
##    Min. 1st Qu.  Median    Mean 3rd Qu.    Max. 
##   29.78   36.14   43.18  121.47   70.46 2567.54
```

How many SNPs have been eliminated with checking the weakness: 0
SNP

### RUN an initial MR:

Initial MR analysis


| id.exposure | id.outcome | outcome | exposure | method | nsnp | b | se | pval |
| --- | --- | --- | --- | --- | --- | --- | --- | --- |
| oUw2e5 | daasDf | outcome | exposure | MR Egger | 84 | -0.0418438 | 0.0854867 | 0.6258107 |
| oUw2e5 | daasDf | outcome | exposure | Weighted median | 84 | 0.0722727 | 0.0615507 | 0.2403161 |
| oUw2e5 | daasDf | outcome | exposure | Inverse variance weighted | 84 | 0.0122784 | 0.0543298 | 0.8212032 |
| oUw2e5 | daasDf | outcome | exposure | Simple mode | 84 | 0.0189745 | 0.1488162 | 0.8988507 |
| oUw2e5 | daasDf | outcome | exposure | Weighted mode | 84 | 0.0498572 | 0.0563517 | 0.3788469 |

Heterogeneity testing


| id.exposure | id.outcome | outcome | exposure | method | Q | Q\_df | Q\_pval |
| --- | --- | --- | --- | --- | --- | --- | --- |
| oUw2e5 | daasDf | outcome | exposure | MR Egger | 198.4561 | 82 | 0 |
| oUw2e5 | daasDf | outcome | exposure | Inverse variance weighted | 200.0878 | 83 | 0 |

pleiotropy testing


| id.exposure | id.outcome | outcome | exposure | egger\_intercept | se | pval |
| --- | --- | --- | --- | --- | --- | --- |
| oUw2e5 | daasDf | outcome | exposure | 0.0018081 | 0.0022021 | 0.413966 |

### Testing Outlier with PRESSO test

```
## [1] "Two SNPs (rs12317268 and rs7439366) were detected by MRPRESSO and excluded for further analyses"
```

MR analysis after excluding SNPs detected by MRPRESSO


| id.exposure | id.outcome | outcome | exposure | method | nsnp | b | se | pval |
| --- | --- | --- | --- | --- | --- | --- | --- | --- |
| oUw2e5 | daasDf | outcome | exposure | MR Egger | 82 | -0.0185637 | 0.0754185 | 0.8062026 |
| oUw2e5 | daasDf | outcome | exposure | Weighted median | 82 | 0.0730557 | 0.0625990 | 0.2431929 |
| oUw2e5 | daasDf | outcome | exposure | Inverse variance weighted | 82 | 0.0507501 | 0.0486693 | 0.2970623 |
| oUw2e5 | daasDf | outcome | exposure | Simple mode | 82 | 0.0252830 | 0.1602860 | 0.8750568 |
| oUw2e5 | daasDf | outcome | exposure | Weighted mode | 82 | 0.0432513 | 0.0584660 | 0.4615790 |

Heterogeneity testing


| id.exposure | id.outcome | outcome | exposure | method | Q | Q\_df | Q\_pval |
| --- | --- | --- | --- | --- | --- | --- | --- |
| oUw2e5 | daasDf | outcome | exposure | MR Egger | 149.4853 | 80 | 4.1e-06 |
| oUw2e5 | daasDf | outcome | exposure | Inverse variance weighted | 152.1795 | 81 | 2.9e-06 |

pleiotropy testing


| id.exposure | id.outcome | outcome | exposure | egger\_intercept | se | pval |
| --- | --- | --- | --- | --- | --- | --- |
| oUw2e5 | daasDf | outcome | exposure | 0.0023277 | 0.0019385 | 0.2333822 |

### Studentized residuals:

### Radial test

```
## 
## Radial IVW
## 
##                    Estimate  Std.Error   t value  Pr(>|t|)
## Effect (Mod.2nd) 0.05074888 0.04866908 1.0427335 0.2970718
## Iterative        0.05074888 0.04866908 1.0427335 0.2970718
## Exact (FE)       0.05160059 0.03551133 1.4530740 0.1462032
## Exact (RE)       0.05120977 0.05140681 0.9961671 0.3221356
## 
## 
## Residual standard error: 1.371 on 81 degrees of freedom
## 
## F-statistic: 1.09 on 1 and 81 DF, p-value: 0.3
## Q-Statistic for heterogeneity: 152.146 on 81 DF , p-value: 2.936746e-06
## 
##  No significant outliers 
## Number of iterations = 2
```

```
## [1] "No significant outliers"
```

### Cook’s distance

In statistics, Cook’s distance or Cook’s D is a commonly used
estimate of the influence of a data point when performing a
least-squares regression analysis.[1] In a practical ordinary least
squares analysis, Cook’s distance can be used in several ways:

1- To indicate influential data points that are particularly worth
checking for validity.

2- To indicate regions of the design space where it would be good to
be able to obtain more data points.

**It is named after the American statistician R. Dennis Cook,
who introduced the concept in 1977.**

Refernce

```
##         12         20         21         25         29         31         50 
## 0.03515705 0.04136593 0.25701721 0.03762193 0.08422961 0.08033235 0.03836803 
##         67 
## 0.06786057
```

### Run After deleting new outlier: Final Results:

MR analysis after deleting outliers


| id.exposure | id.outcome | outcome | exposure | method | nsnp | b | se | pval |
| --- | --- | --- | --- | --- | --- | --- | --- | --- |
| oUw2e5 | daasDf | outcome | exposure | MR Egger | 70 | 0.2466067 | 0.1387102 | 0.0798986 |
| oUw2e5 | daasDf | outcome | exposure | Weighted median | 70 | 0.2233635 | 0.0822726 | 0.0066292 |
| oUw2e5 | daasDf | outcome | exposure | Inverse variance weighted | 70 | 0.2035989 | 0.0563823 | 0.0003050 |
| oUw2e5 | daasDf | outcome | exposure | Simple mode | 70 | 0.0316973 | 0.1759626 | 0.8575732 |
| oUw2e5 | daasDf | outcome | exposure | Weighted mode | 70 | 0.2194134 | 0.1085214 | 0.0470722 |

Heterogeneity testing


| id.exposure | id.outcome | outcome | exposure | method | Q | Q\_df | Q\_pval |
| --- | --- | --- | --- | --- | --- | --- | --- |
| oUw2e5 | daasDf | outcome | exposure | MR Egger | 86.59380 | 68 | 0.0636779 |
| oUw2e5 | daasDf | outcome | exposure | Inverse variance weighted | 86.74083 | 69 | 0.0731205 |

pleiotropy testing


| id.exposure | id.outcome | outcome | exposure | egger\_intercept | se | pval |
| --- | --- | --- | --- | --- | --- | --- |
| oUw2e5 | daasDf | outcome | exposure | -0.0008971 | 0.00264 | 0.735061 |

### Sensitivity analyses with MendelianRandomization Package

```
## 
## Inverse-variance weighted method
## (variants uncorrelated, random-effect model)
## 
## Number of Variants : 70 
## 
## ------------------------------------------------------------------
##  Method Estimate Std Error 95% CI       p-value
##     IVW    0.204     0.056 0.093, 0.314   0.000
## ------------------------------------------------------------------
## Residual standard error =  1.121 
## Heterogeneity test statistic (Cochran's Q) = 86.7408 on 69 degrees of freedom, (p-value = 0.0731). I^2 = 20.5%.
```

```
##                     Method Estimate Std Error 95% CI        P-value
##              Simple median    0.123     0.080  -0.034 0.280   0.125
##            Weighted median    0.224     0.080   0.068 0.381   0.005
##  Penalized weighted median    0.223     0.080   0.066 0.380   0.005
##                                                                    
##                        IVW    0.204     0.056   0.093 0.314   0.000
##              Penalized IVW    0.204     0.056   0.093 0.314   0.000
##                 Robust IVW    0.193     0.060   0.075 0.310   0.001
##       Penalized robust IVW    0.193     0.060   0.075 0.310   0.001
##                                                                    
##                   MR-Egger    0.247     0.139  -0.025 0.518   0.075
##                (intercept)   -0.001     0.003  -0.006 0.004   0.734
##         Penalized MR-Egger    0.247     0.139  -0.025 0.518   0.075
##                (intercept)   -0.001     0.003  -0.006 0.004   0.734
##            Robust MR-Egger    0.276     0.117   0.046 0.507   0.018
##                (intercept)   -0.002     0.003  -0.007 0.003   0.486
##  Penalized robust MR-Egger    0.276     0.117   0.046 0.507   0.018
##                (intercept)   -0.002     0.003  -0.007 0.003   0.486
```

| id.exposure | id.outcome | exposure | outcome | snp\_r2.exposure | snp\_r2.outcome | correct\_causal\_direction | steiger\_pval |
| --- | --- | --- | --- | --- | --- | --- | --- |
| oUw2e5 | daasDf | exposure | outcome | 0.0109999 | 0.0020872 | TRUE | 0 |

```
## $r2_exp
## [1] 0
## 
## $r2_out
## [1] 0.25
## 
## $r2_exp_adj
## [1] 0
## 
## $r2_out_adj
## [1] 0.25
## 
## $correct_causal_direction
## [1] FALSE
## 
## $steiger_test
## [1] 0
## 
## $correct_causal_direction_adj
## [1] FALSE
## 
## $steiger_test_adj
## [1] 0
## 
## $vz
## [1] NaN
## 
## $vz0
## [1] 0
## 
## $vz1
## [1] NaN
## 
## $sensitivity_ratio
## [1] NaN
## 
## $sensitivity_plot
```

### Working with MRraps

```
## $beta.hat
## [1] 0.2075324
## 
## $beta.se
## [1] 0.05105289
## 
## $beta.p.value
## [1] 4.80227e-05
## 
## $naive.se
## [1] 0.05066738
## 
## $chi.sq.test
## [1] 86.42346
```

```
##   over.dispersion loss.function  beta.hat    beta.se
## 1           FALSE            l2 0.2075324 0.05105289
## 2           FALSE         huber 0.1909614 0.05234920
## 3           FALSE         tukey 0.1939161 0.05235445
## 4            TRUE            l2 0.2049852 0.05640578
## 5            TRUE         huber 0.1923723 0.05880302
## 6            TRUE         tukey 0.1963334 0.05908604
```

```
## 
## Constrained maximum likelihood method (MRcML) 
## Number of Variants:  70 
## Results for:  cML-MA-BIC 
## ------------------------------------------------------------------
##      Method Estimate    SE Pvalue        95% CI
##  cML-MA-BIC    0.207 0.051  0.000 [0.107,0.307]
## ------------------------------------------------------------------
```

```
## 
## Debiased inverse-variance weighted method
## (Over.dispersion:TRUE)
## 
## Number of Variants : 70 
## ------------------------------------------------------------------
##  Method Estimate Std Error 95% CI       p-value Condition
##    dIVW    0.207     0.057 0.095, 0.319   0.000   540.818
## ------------------------------------------------------------------
```

```
## 
## Mode-based method of Hartwig et al
## (weighted, delta standard errors [not assuming NOME], bandwidth factor = 1)
## 
## Number of Variants : 70 
## ------------------------------------------------------------------
##  Method Estimate Std Error 95% CI       p-value
##     MBE    0.219     0.081 0.060, 0.379   0.007
## ------------------------------------------------------------------
```

## [VitD and TSH]

### Introduction

- **Title:** Investigating the causality between
  **25-Hydroxyvitamin D level on TSH**

  - Exposure: 25-Hydroxyvitamin D level, Data downloaded from GWAS catalog
    Database | Reference paper: Joana A. Revez
    Paper 2020 | GWAS ID: GCST90000616
  - **Sample size**: 417,580
  - Outcome: TSH, Data downloaded from Thyroid
    Omics Consortium | Reference paper: Alexander
    Teumer 2018 | GWAS ID: phs000930
  - **Sample size**:54,288
  - Ancestry: **European**

### Data Preparation

1- **Number of total SNPs in exposure:** 7,250,104
SNPs

2- **Number of SNPs exposure with p-value <** \(5 \times 10^-8\): 16,012 SNPs

3- **Number of SNPs exposure after clumping** : 115
SNPs

4- **Number of total SNPs in outcome**: 7,742,681
SNPs

5- **Number of common variants between exposure and
outcome**: 101 SNPs

6- **Number of SNPs after harmonization (action=3)**  =
84 SNPs

7- **Number of SNPs after removing HLA region with exploring in
HLA Genes,
Nomenclature** = 84 SNP

8- **Number of SNPs after removing those that have MAF <
0.01** = 84 SNPs

10- Checking pleiotropy by PhenoScanner:

How many SNPs have been eliminated after checking the PhenoScanner
website: 1 SNP (rs73413596)

### Checking weakness of the instruments

```
##    Min. 1st Qu.  Median    Mean 3rd Qu.    Max. 
##   29.78   36.14   43.24  122.48   70.86 2567.54
```

How many SNPs have been eliminated with checking the weakness: 0
SNP

### RUN an initial MR:

Initial MR analysis


| id.exposure | id.outcome | outcome | exposure | method | nsnp | b | se | pval |
| --- | --- | --- | --- | --- | --- | --- | --- | --- |
| qxmOS6 | 7CyyhT | outcome | exposure | MR Egger | 83 | -0.0282157 | 0.0675033 | 0.6770598 |
| qxmOS6 | 7CyyhT | outcome | exposure | Weighted median | 83 | -0.0145698 | 0.0579419 | 0.8014616 |
| qxmOS6 | 7CyyhT | outcome | exposure | Inverse variance weighted | 83 | -0.0469732 | 0.0427422 | 0.2717736 |
| qxmOS6 | 7CyyhT | outcome | exposure | Simple mode | 83 | -0.0636091 | 0.1136336 | 0.5771597 |
| qxmOS6 | 7CyyhT | outcome | exposure | Weighted mode | 83 | -0.0429973 | 0.0487954 | 0.3807988 |

Heterogeneity testing


| id.exposure | id.outcome | outcome | exposure | method | Q | Q\_df | Q\_pval |
| --- | --- | --- | --- | --- | --- | --- | --- |
| qxmOS6 | 7CyyhT | outcome | exposure | MR Egger | 138.6384 | 81 | 7.07e-05 |
| qxmOS6 | 7CyyhT | outcome | exposure | Inverse variance weighted | 138.8606 | 82 | 8.97e-05 |

pleiotropy testing


| id.exposure | id.outcome | outcome | exposure | egger\_intercept | se | pval |
| --- | --- | --- | --- | --- | --- | --- |
| qxmOS6 | 7CyyhT | outcome | exposure | -0.0006249 | 0.0017343 | 0.7195554 |

### Testing Outlier with PRESSO test

```
## [1] "One SNP (rs532436) was detected by MRPRESSO and excluded for further analyses"
```

MR analysis after excluding SNPs detected by MRPRESSO


| id.exposure | id.outcome | outcome | exposure | method | nsnp | b | se | pval |
| --- | --- | --- | --- | --- | --- | --- | --- | --- |
| qxmOS6 | 7CyyhT | outcome | exposure | MR Egger | 82 | -0.0331682 | 0.0543454 | 0.5433790 |
| qxmOS6 | 7CyyhT | outcome | exposure | Weighted median | 82 | -0.0145490 | 0.0565448 | 0.7969470 |
| qxmOS6 | 7CyyhT | outcome | exposure | Inverse variance weighted | 82 | -0.0295222 | 0.0344758 | 0.3918233 |
| qxmOS6 | 7CyyhT | outcome | exposure | Simple mode | 82 | -0.0579555 | 0.1182083 | 0.6252593 |
| qxmOS6 | 7CyyhT | outcome | exposure | Weighted mode | 82 | -0.0372654 | 0.0526553 | 0.4811487 |

Heterogeneity testing


| id.exposure | id.outcome | outcome | exposure | method | Q | Q\_df | Q\_pval |
| --- | --- | --- | --- | --- | --- | --- | --- |
| qxmOS6 | 7CyyhT | outcome | exposure | MR Egger | 88.73302 | 80 | 0.2360567 |
| qxmOS6 | 7CyyhT | outcome | exposure | Inverse variance weighted | 88.74145 | 81 | 0.2604797 |

pleiotropy testing


| id.exposure | id.outcome | outcome | exposure | egger\_intercept | se | pval |
| --- | --- | --- | --- | --- | --- | --- |
| qxmOS6 | 7CyyhT | outcome | exposure | 0.0001221 | 0.0014005 | 0.9307676 |

### Studentized residuals:

### Radial test

```
## 
## Radial IVW
## 
##                     Estimate  Std.Error    t value  Pr(>|t|)
## Effect (Mod.2nd) -0.02952200 0.03447575 -0.8563121 0.3918252
## Iterative        -0.02952200 0.03447575 -0.8563121 0.3918252
## Exact (FE)       -0.02979675 0.03293912 -0.9046008 0.3656769
## Exact (RE)       -0.02979331 0.03191391 -0.9335525 0.3533094
## 
## 
## Residual standard error: 1.047 on 81 degrees of freedom
## 
## F-statistic: 0.73 on 1 and 81 DF, p-value: 0.394
## Q-Statistic for heterogeneity: 88.73384 on 81 DF , p-value: 0.2606622
## 
##  No significant outliers 
## Number of iterations = 2
```

```
## [1] "No significant outliers"
```

### Sensitivity analyses with MendelianRandomization Package

```
## 
## Inverse-variance weighted method
## (variants uncorrelated, random-effect model)
## 
## Number of Variants : 82 
## 
## ------------------------------------------------------------------
##  Method Estimate Std Error  95% CI       p-value
##     IVW   -0.030     0.034 -0.097, 0.038   0.392
## ------------------------------------------------------------------
## Residual standard error =  1.047 
## Heterogeneity test statistic (Cochran's Q) = 88.7414 on 81 degrees of freedom, (p-value = 0.2605). I^2 = 8.7%.
```

```
##                     Method Estimate Std Error 95% CI        P-value
##              Simple median   -0.031     0.059  -0.145 0.084   0.602
##            Weighted median   -0.015     0.055  -0.123 0.094   0.792
##  Penalized weighted median   -0.015     0.056  -0.123 0.094   0.794
##                                                                    
##                        IVW   -0.030     0.034  -0.097 0.038   0.392
##              Penalized IVW   -0.030     0.034  -0.097 0.038   0.392
##                 Robust IVW   -0.028     0.032  -0.090 0.034   0.383
##       Penalized robust IVW   -0.028     0.032  -0.090 0.034   0.383
##                                                                    
##                   MR-Egger   -0.033     0.054  -0.140 0.073   0.542
##                (intercept)    0.000     0.001  -0.003 0.003   0.931
##         Penalized MR-Egger   -0.033     0.054  -0.140 0.073   0.541
##                (intercept)    0.000     0.001  -0.003 0.003   0.930
##            Robust MR-Egger   -0.032     0.049  -0.128 0.063   0.507
##                (intercept)    0.000     0.001  -0.003 0.003   0.910
##  Penalized robust MR-Egger   -0.032     0.049  -0.128 0.063   0.507
##                (intercept)    0.000     0.001  -0.003 0.003   0.910
```

| id.exposure | id.outcome | exposure | outcome | snp\_r2.exposure | snp\_r2.outcome | correct\_causal\_direction | steiger\_pval |
| --- | --- | --- | --- | --- | --- | --- | --- |
| qxmOS6 | 7CyyhT | exposure | outcome | 0.0192884 | 0.0016496 | TRUE | 0 |

```
## $r2_exp
## [1] 0
## 
## $r2_out
## [1] 0.25
## 
## $r2_exp_adj
## [1] 0
## 
## $r2_out_adj
## [1] 0.25
## 
## $correct_causal_direction
## [1] FALSE
## 
## $steiger_test
## [1] 0
## 
## $correct_causal_direction_adj
## [1] FALSE
## 
## $steiger_test_adj
## [1] 0
## 
## $vz
## [1] NaN
## 
## $vz0
## [1] 0
## 
## $vz1
## [1] NaN
## 
## $sensitivity_ratio
## [1] NaN
## 
## $sensitivity_plot
```

### Working with MRraps

```
## $beta.hat
## [1] -0.02980411
## 
## $beta.se
## [1] 0.03321019
## 
## $beta.p.value
## [1] 0.3694849
## 
## $naive.se
## [1] 0.03306716
## 
## $chi.sq.test
## [1] 88.73377
```

```
##   over.dispersion loss.function    beta.hat    beta.se
## 1           FALSE            l2 -0.02980411 0.03321019
## 2           FALSE         huber -0.02894143 0.03407276
## 3           FALSE         tukey -0.02726968 0.03407253
## 4            TRUE            l2 -0.03147601 0.03461906
## 5            TRUE         huber -0.03107808 0.03698782
## 6            TRUE         tukey -0.03009628 0.03673932
```

```
## 
## Constrained maximum likelihood method (MRcML) 
## Number of Variants:  82 
## Results for:  cML-MA-BIC 
## ------------------------------------------------------------------
##      Method Estimate    SE Pvalue         95% CI
##  cML-MA-BIC   -0.030 0.033  0.371 [-0.094,0.035]
## ------------------------------------------------------------------
```

```
## 
## Debiased inverse-variance weighted method
## (Over.dispersion:TRUE)
## 
## Number of Variants : 82 
## ------------------------------------------------------------------
##  Method Estimate Std Error  95% CI       p-value Condition
##    dIVW   -0.030     0.034 -0.097, 0.038   0.388  1108.127
## ------------------------------------------------------------------
```

```
## 
## Mode-based method of Hartwig et al
## (weighted, delta standard errors [not assuming NOME], bandwidth factor = 1)
## 
## Number of Variants : 82 
## ------------------------------------------------------------------
##  Method Estimate Std Error  95% CI       p-value
##     MBE   -0.037     0.059 -0.153, 0.079   0.529
## ------------------------------------------------------------------
```
